# Coastal Wetlands Drive Isotopic Niche Plasticity of Top Predator Fish Communities in Green Bay, Lake Michigan (USA)

**DOI:** 10.1002/ece3.71463

**Published:** 2025-05-23

**Authors:** Tania V. Rojas, Katherine E. O'Reilly, Christopher J. Houghton, Jeremiah S. Shrovnal, Martin B. Berg, Donald G. Uzarski, Gary A. Lamberti, Patrick S. Forsythe

**Affiliations:** ^1^ Aquatic Ecology and Fisheries Laboratory, Department of Natural and Applied Sciences University of Wisconsin‐Green Bay Green Bay Wisconsin USA; ^2^ Department of Biology University of Kentucky Lexington Kentucky USA; ^3^ Illinois‐Indiana Sea Grant Champaign Illinois USA; ^4^ Wisconsin Department of Natural Resources Bureau of Fisheries Management Bayfield Wisconsin USA; ^5^ Department of Biology Loyola University Chicago Chicago Illinois USA; ^6^ Institute for Great Lakes Research Central Michigan University Mount Pleasant Michigan USA; ^7^ Department of Biological Sciences University of Notre Dame Notre Dame Indiana USA

**Keywords:** community metrics, food webs, Great Lakes, sport fish, stable isotopes, trophic structure

## Abstract

Green Bay, the largest freshwater embayment in Lake Michigan, is a unique environment consisting of a trophic gradient along its north‐to‐south axis that shapes the heterogeneous and dynamic habitat, driving diverse fish behavior among the remnant coastal wetlands of Green Bay. Although previous studies of aquatic food webs in Green Bay have focused on lower trophic levels to estimate trophic shift responses, we examined trophic relationships among fish communities in five coastal wetland areas of Green Bay, emphasizing top predator species of recreational and commercial importance in Lake Michigan. We used stable isotope‐based community metrics and Bayesian mixing models to describe food web structure and patterns in trophic position, isotopic niche, and diet contributions of top predators, including bowfin *Amia calva* , largemouth bass *Micropterus salmoides* , northern pike *Esox lucius* , smallmouth bass *Micropterus dolomieu* , walleye *Sander vitreus* , and yellow perch *Perca flavescens* . We found high probability (> 70%) of overlap among the isotopic niches of piscivorous, invertivorous, and benthivorous fish, reflecting the capacity of different feeding guilds to exploit isotopically similar sources. In addition, we found that invertivorous fish represented a critical trophic link between the top‐level fish populations and lower levels, such as aquatic invertebrates. Lastly, we found that top predators diversified their diet in lacustrine wetlands but had a distinct foraging habitat preference in riverine wetlands, emphasizing the importance of habitat type and structure in feeding diversity. Top predators in Green Bay displayed a high degree of isotopic niche plasticity, as evidenced by differences in trophic positions and foraging strategies at each site. Flexibility in fish feeding ecology, such as variations in dietary overlap and niche space, along with the hydrogeomorphic setting, underpins the ability of fish communities of Green Bay to thrive under different stressors.

## Graphical abstract

Green Bay, the largest freshwater embayment in Lake Michigan, is a unique environment that drives diverse fish behavior among their remnant coastal wetlands. We used Bayesian models to describe the food web structure of top predators in Green Bay. Our findings suggested significant isotopic niche overlap among piscivorous fish and other feeding guilds, along with diet diversification between aquatic invertebrates and forage fish in lacustrine and riverine wetlands.

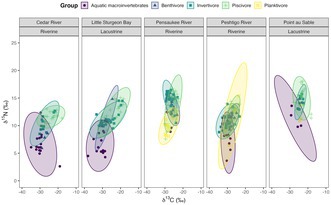

## 1 Introduction

Coastal wetlands cover approximately 6% of the Earth's surface (An and Verhoeven 2019; Mitsch and Gosselink 2015) and are characterized by a permanent or seasonal connection to fresh waters that leads to complicated predator–prey relationships and dynamic energy transfer among trophic levels (Keough et al. 1999; Osmundson et al. 2002). Coastal wetlands also provide diverse habitats for terrestrial and aquatic species (Cvetkovic and Chow‐Fraser 2011), such as fish species that use wetland habitats for spawning and as nursery and/or forage areas (Jude and Pappas 1992; O'Reilly et al. 2023).

In the Laurentian Great Lakes (hereafter, the Great Lakes), coastal wetlands are characterized by hydrologic connections to the lake and the presence of submerged, floating, and emerging macrophytes (e.g., *Typha* spp.) and structure and function are determined by water‐level fluctuations (Frieswyk and Zedler 2007; Johnston et al. 2007). These wetlands also provide habitat for up to 80% of the species of fish in the Great Lakes during their life cycle (Chow‐Fraser and Albert 1999; Goforth and Carman 2005; Uzarski et al. 2005). In the last century, Great Lakes coastal wetland area has declined by 60%–75%, and the remaining wetlands face ongoing degradation (Bosley 1978; Brazner 1997) due to urban development, invasive species, chemical pollution, and land use practices that increase turbidity and impact dissolved oxygen concentrations, pH, alkalinity, and specific conductance (Burdon et al. 2020; Olokotum et al. 2020; Trebitz et al. 2007; Uzarski et al. 2017).

Green Bay, located in northwestern Lake Michigan (USA), is the largest freshwater estuary in the world (Smith et al. 1988) and harbors about 22% of the coastal wetlands that remain in Lake Michigan (Harris et al. 2018; Krieger et al. 1992). The water chemistry of Green Bay is characterized by a relatively unique ecological gradient influenced by the prevailing hydrodynamics due to eastern‐to‐western circulation patterns, which are stronger during the summer (Hamidi et al. 2015), and large inputs of nutrients and sediments from the Fox River and Menominee River watersheds (Mooney et al. 2020; Son and Wang 2019). These processes collectively produce a gradient consisting of eutrophic south‐to‐meso‐oligotrophic northern open waters (King and Brazner 1999). The trophic gradient in Green Bay contributes to structurally complex coastal wetlands with distinct patterns of fish assemblages in coastal habitats from south to north (Brazner and Beals 1997).

Previous studies have shown a high fish utilization of coastal wetlands in Green Bay promoted by their connection to Lake Michigan (Jude and Pappas 1992), particularly as nursery habitat for both resident and migratory species (Brazner 1997). Green Bay provides habitat for more than 95% of walleye ( *Sander vitreus* ) harvested in the Lake Michigan recreational fishery, in addition to other regionally important species, such as northern pike ( *Esox lucius* ) and yellow perch ( *Perca flavescens* ) (Zorn and Kramer 2022). Resident species interact with coastal wetlands for nursery and foraging (Brazner et al. 2000), whereas migratory species visit coastal wetlands periodically. Piscivorous (i.e., predatory) fish spawn primarily in wetlands and use them as a nursery area (Jude and Pappas 1992). For instance, northern pike use emergent vegetation for egg deposition and fry growth and survival (Casselman and Lewis 1996). During their early life stages, predatory fish also influence the composition of the community of aquatic food webs by exerting top‐down control of invertebrate assemblages via prey selection (Krabbenhoft et al. 2017).

The coastal wetland area in Green Bay has declined by 30%–60% since the early 1800s due to human and natural disturbances that have altered not only wetland vegetation but also the composition of fish species (Bosley 1978). Trawl surveys in recent decades have shown a decline in fish populations in northern Green Bay due to environmental changes (e.g., water clarity and temperature), anthropogenic drivers (e.g., invasive species), and changes in trophic interactions (e.g., declines in forage fish) (Zorn and Kramer 2022). Although abiotic factors such as water levels may impact the production of top predators such as northern pike, walleye, and smallmouth bass ( *Micropterus dolomieu* ) due to habitat availability, these trophic interactions in relation to hydrology remain understudied in the coastal habitats of Green Bay.

Food web analyses are used to understand trophic interactions between consumers and resources (Sierszen et al. 2006) through the characterization of trophic niches (Elton 1927). Within this scope, stable isotope analysis (SIA) has been a useful tool to describe the trophic structure and estimate the isotopic niche (a measure of the trophic niche [Bearhop et al. 2004]) of an organism to a community level, characterize trophic diversity and redundancy (Layman et al. 2007), and uncover aspects of fish behavior, such as diet and habitat use (Church 2012; Fry 1988) or ontogenetic diet shifts (Gopalan et al. 1998; Turschak and Bootsma 2015). Isotopic niches have been widely studied to understand the adaptation of organisms to changes in environmental conditions, such as biological invasions in the Great Lakes (Guzzo et al. 2013; Pettitt‐Wade et al. 2015). Changes to the size of isotopic niche spaces can indicate changes in resource availability (Pool et al. 2017). The diversity of the isotopic niche breadth and width, combined with overlaps, thus provides insights into different functional niche mechanisms. Furthermore, studying ontogenetic diet changes (i.e., changes in diet or habitat) helps to elucidate fish utilization of available resources that are crucial for young‐of‐year (YOY) survival and recruitment (Gopalan et al. 1998).

The regional and site‐specific conditions of the coastal wetlands of Green Bay (Brazner and Beals 1997) have led to fish assemblages whose interactions remain poorly understood. To date, food web studies in Green Bay have typically focused on assessing lower trophic levels, such as phytoplankton, zooplankton, and macroinvertebrate communities (King and Brazner 1999; Sager and Richman 1991; Schneider and Sager 2007; Sierszen et al. 2012), and few studies have examined the food web pathways to top levels (i.e., fish communities). For instance, in other areas of the Great Lakes, Turschak and Bootsma (2015) and Guzzo et al. (2013) found that changes in basal resources could affect niche diversification and the spatial distribution of species, such as white and yellow perch, leading to shifts in trophic structure and niche (i.e., trophic niche plasticity). These knowledge gaps for Green Bay are considerable in terms of fish behavior and trophic ecology (e.g., niche shifts and foraging mechanisms) and in our overall understanding of coastal wetland ecosystem functioning (Harris et al. 1987, 2018; McDonald‐Madden et al. 2016).

Our study aims to evaluate the variation in the trophic structure of predatory fish (hereafter “top predators”) of different coastal wetland ecotypes in Green Bay. We focus on six predatory fish species—bowfin ( *Amia calva* ), largemouth bass ( *Micropterus salmoides* ), northern pike, smallmouth bass, walleye, and yellow perch—that contribute to the recreational and commercial fishing in Lake Michigan. We compared stable isotope‐derived metrics of the trophic structure of top predator communities and evaluated how energy pathways responded to changes in resource abundance and diversity. By examining the variation in resource use and potential distinct isotopic communities (Eberts et al. 2017), we predicted that (1) the trophic features of piscivorous fish communities and the foraging behavior of the top predators are different in all sites and (2) the isotopic niches of top predator vary in all sites regardless of type of wetland habitat or resource availability.

## 2 Site Description

**FIGURE 1:**
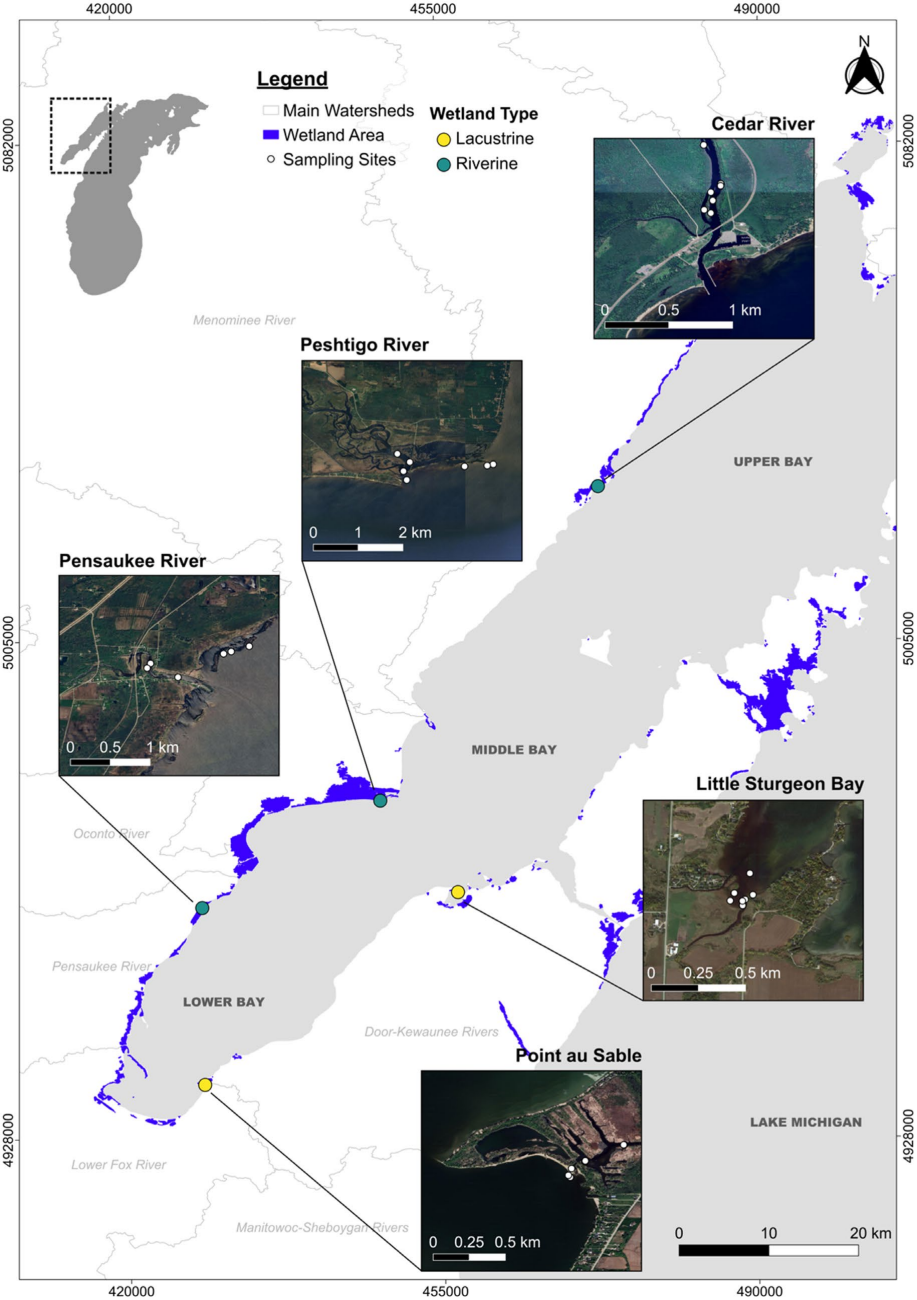
Map of sampled coastal wetland sites and hydrogeomorphic characterization based on Albert et al. (2005). Wetland areas (blue shading) were obtained from the Great Lakes Coastal Wetland Consortium mapping tool (https://www.greatlakeswetlands.org/Map.vbhtml#). Watershed delimitations were obtained from the Wisconsin and Michigan Departments of Natural Resources.

We sampled five coastal wetlands within Green Bay: Cedar River, Little Sturgeon Bay, Pensaukee River, Peshtigo River, and Point au Sable (Figure 1). Wetlands were chosen to reflect the diversity of the riverine and lacustrine systems outlined previously (Albert et al. 2005; Brinson 1993; Keough et al. 1999). Selected wetlands also represented the latitudinal gradient of Green Bay to capture the broadest diversity of wetlands related to fish communities and supported the fish production of Lake Michigan (Brazner 1997).

In Upper Green Bay, Cedar River is a riverine wetland characterized by an open drowned river‐mouth ecotype that receives direct surface water from both the lake and the river. This wetland has variable inorganic substrate (from clay to gravel) and contains biota tolerant of flooding and high sediment concentrations.

In Middle Green Bay, Little Sturgeon Bay is a lacustrine wetland characterized by an unrestricted bay formed by a successional barrier beach lagoon strongly influenced by lake‐water fluctuations such that vegetation was adapted to hydraulic stress and aligned behind barrier sand bars. Pensaukee River is a riverine wetland characterized by an open shoreline that received direct surface water and was therefore highly influenced by the elevation of the lake. Peshtigo River is a riverine wetland characterized by a drowned river mouth ecotype consisting of a delta and braided channels. Although the main channel was directly connected to the lake, in which the secondary channels were seasonally connected and had a relatively uniform inorganic substrate of sand and gravel and a high sediment supply.

In Lower Green Bay, Point au Sable is an enclosed lacustrine wetland characterized by a sand‐spit embayment with inland water bodies seasonally connected to the lake. This ecotype is the result of sandy sediment transport that is not impeded by any barrier.

## 3 Materials and Methods

### 3.1 Sample Collection and Processing

Wetlands were sampled for two summers (June–August of 2014 and 2015) to provide temporal replication and avoid seasonal bias. Fish and benthic macroinvertebrate samples for stable isotope analysis were collected within the wetland and from nearby river mouths to target representative habitats (Figure 1). All biological sampling protocols were considered standard procedures as part of the Great Lakes Coastal Wetland Monitoring Program (GLCWMP) (Uzarski et al. 2017) and followed standard methods for fisheries surveys (Hoffman et al. 2010; Osmundson et al. 2002). All samples were collected following institutional procedures of the American Association for Laboratory Animal Science (IACUC).

At each wetland site, D‐frame dip nets with 0.5 mm mesh were used to collect benthic macroinvertebrates [e.g., snails (Gastropoda), mayflies (Baetidae and Caenidae), midge larvae (Chironomidae), amphipods (*Hyalella* and *Gammarus*), damselfly and dragonfly immatures (Odonata)]. In general, nets were swept through existing wetland vegetation and along the surface of the sediments to sample both dominant habitats. Invertebrates were then field‐picked from the net contents and stored in cleaned glass vials before being placed on ice. Our goal is to collect enough replicate samples per taxon. Wetland fish were collected using a combination of 5–10 large (4′ × 3′) and small (1.5′ × 3′) modified fyke nets (Uzarski et al. 2005) set for one net night in shallow waters (< 2 m depth). Fish collection was supplemented using boat electrofishing for an appropriate amount of time to capture enough individuals for isotope analyses. Please note that sample sizes for walleye and northern pike were relatively low due to capturing limitations. Samples were then stored on ice, transported back to the laboratory, and frozen at −20°C.

Collected fishes were identified by species and the total length was measured, and each was classified according to the published feeding ecology by species. Feeding guilds were primarily obtained from Becker (1983) with additional characterizations from Gamble et al. (2011), Keast (1985), Malinowski et al. (2022), and Reid et al. (2009). Collected top predators were separated by age (i.e., adults and YOY) based on total length due to differences in functional ecology due to ontogenetic diet shifts, that is, shifts to piscivore with increasing body size (indicated as length threshold in Figure 3). Although acknowledging that YOY fishes can quickly transition between feeding guilds as they grow, all YOY fish were considered “prey” in subsequent analyses for simplicity. In addition, aquatic invertebrate orders were grouped to distinguish different resource assemblages for food web interpretation and mixing model analyses (Peel et al. 2019).

### 3.2 Stable Isotope Analysis (SIA)

Samples for isotope analysis were rinsed with E‐pure water; for fish, only dorsal muscle was used, and macroinvertebrates were removed from the shells or cases when present. All macroinvertebrate and fish samples were oven dried at 70°C for 48 h and homogenized using a ceramic mortar and pestle. Samples were analyzed using a Thermo‐Finnigan Delta Plus isotope ratio mass spectrometer (IRMS) located at the Center for Environmental Science and Technology (CEST) at the University of Notre Dame. Samples for this analysis were selected to maximize the range of sizes from YOY to adult individuals per species. Detailed information on the sample analyses can be found in O'Reilly et al. (2023).

The isotopic composition was denoted in delta (δ) notation, that is, differences between the isotopic ratios in the samples and in international standards (VPBD for δ^13^C and atmospheric nitrogen for δ^15^N). The observed analytical precisions of δ^13^C and δ^15^N based on replicates of a laboratory protein standard included with every instrument run were ±0.33% and ±0.22%, respectively. To avoid bias resulting from chemical interference in isotopic compositions, we normalized the δ^13^C for fauna with C:N>3.5 using the following formula (Post et al. 2007):

$$\delta^{13}C_{\text{normalized}}=\delta^{13}C_{\text{untreated}}-3.32+0.99\times C:N$$

We also used the aquatic macroinvertebrates (i.e., primary consumers) as a proxy of site‐specific δ^13^C and δ^15^N habitat baselines to effectively integrate spatial and temporal isotopic variation in the trophic position of top predators (Vander Zanden and Rasmussen 1999). ANOVA tests showed no differences in primary consumers and fish isotopic compositions within wetland ecotypes between sampling years. Therefore, we pooled data from both years for all further analyses. In addition, our preliminary analysis (unpublished data) did not show significant differences between the trophic positions of top predators between wetland and nearshore habitats.

Normality of stable isotope data was evaluated using the Shapiro–Wilk normality test. Additionally, we generated δ^13^C and δ^15^N stable isotope biplots at each study site with a 95% probability region and estimated the overlap of the isotopic niche between the top predators and feeding guilds using the package “nicheROVER” (Swanson et al. 2015). All analyses were performed using R version 4.2.0 (R Core Team 2022).

### 3.3 Trophic Position

The trophic position of predatory fish was calculated using dorsal muscle tissue δ^15^N data and a baseline correction method (McCutchan et al. 2003) at all sites. The following formula interprets the fish δ^15^N value relative to that of the site‐specific baseline (i.e., primary consumer) δ^15^N value (Vander Zanden and Rasmussen 1999):

$$\text{Trophic position}=\left(\frac{\delta^{15}N_{\text{consumer}}-\delta^{15}N_{\text{base}}}{∆\delta^{15}N}\right)+2$$

$\delta^{15}N_{\text{base}}$ is the mean value of the δ^15^N of primary consumers (i.e., aquatic macroinvertebrates) and $∆\delta^{15}N$ shows the increase in δ^15^N per trophic level, which was assumed to be 3.4 (McCutchan et al. 2003). Note that trophic position was 1 for primary producers, 2 for primary consumers, and so on.

To facilitate a more accurate comparison of trophic positions across sites, we removed invertebrate data points that do not represent primary consumers when calculating baseline values. We excluded taxa such as Odonata where the trophic role was ambiguous. This adjustment ensured that baseline values accurately reflect isotopic signatures of true primary consumers.

Trophic positions of the consumers were analyzed with one‐way analysis of variance (ANOVA) to test for significant differences in the trophic position of the same species among sites. The Tukey–Kramer HSD (Honestly Significant Difference) procedure was used to test for differences among sites as well. Statistical analyses were performed in R version 4.2.0 (R Core Team 2022).

### 3.4 Community Metrics and Isotopic Niche

We compared the trophic structure for each wetland site by calculating community metrics based on observed δ^13^C and δ^15^N values. Metrics were obtained using the means of samples for each trophic group (i.e., organisms that have a similar feeding behavior) to represent community‐wide aspects of trophic structure, such as trophic diversity and trophic redundancy (Layman et al. 2007). Trophic diversity at each site was estimated by comparing the values of the total extent of spacing within biplot spaces using three metrics: δ^15^N range (NR), δ^13^C range (CR), and mean distance to centroid (CD). NR represents the vertical structure or trophic length of the community, CR indicates the diversity of the basal resources, and CD informs on niche width, the spacing of species, and the average degree of trophic diversity (Layman et al. 2007). Trophic redundancy was estimated based on two metrics: (1) the relative position of each species with others within the niche space using the mean nearest neighbor distance (NND) to measure the overall density of the species packing, and (2) the standard deviation of the nearest neighbor distance (SDNND) to estimate the evenness of the species packing and indicates how diverse trophic niches are (Donázar‐Aramendía et al. 2019; Layman et al. 2007). In addition, total area or convex hull (TA) represents the total amount of isotopic niche space occupied by or the extent of trophic diversity within a food web.

Bayesian standard ellipse areas (SEA) provided a comparable description of the isotopic niche of a single organism or community (Jackson et al. 2011). We calculated the corrected standard ellipse area (SEA_C_) for the top predator communities using the R package SIBER to calculate community‐based metrics under the routine of SIAR (Jackson et al. 2011; Parnell et al. 2013). This method propagates uncertainty in the mean using Bayesian inference (SEA_B_), allowing a direct comparison between community members and their isotopic niche avoiding the bias at small sample sizes (Jackson et al. 2011). Only species with n≥3 at each site were included in the analyses to avoid underestimation in the calculation of the means and the covariance matrix and not exceed degrees of freedom (Jackson et al. 2011). All statistical analyses were performed in R version 4.2.0 (R Core Team 2022).

### 3.5 Mixing Models for Diet Contributions

We applied Bayesian mixing models to estimate the relative contribution of prey resources to the top predator for both overall populations and separately by species. For each model per site, end members (or sources) were the prey fish (invertivorous, benthivorous, and planktivorous fish) and the benthic macroinvertebrates. Top predators by species—bowfin, largemouth bass, northern pike, smallmouth bass, walleye, and yellow perch—were included in the models as a fixed effect to assess whether the proportions of diet differed among species and sites. We then used the medians, that is, 50% quantile, to compare the variation in diet for each species or population, thus allowing us to compare output estimates with the minimum expected absolute error since posterior means are less influenced by tails of the distribution as suggested by Stock et al. (2018).

To improve the accuracy of our mixing model estimates, we followed the recommendations to perform pre‐model diagnostics before applying mixing models by Phillips et al. (2014) and Smith et al. (2013). We removed consumer data points that fell outside of the mixing region, that is, consumers with very low probability (< 5%) (Smith et al. 2013). Raw mixing regions are included in Figures S1–S5. This step aimed to reduce the likelihood of extrapolating beyond the model's assumptions and supports our isotopic inferences.

Models were run separately for each site using the R package “MixSIAR” (Stock et al. 2018; Stock and Semmens 2016) in which potential prey sources included benthivorous, invertivorous, and planktivorous fish, along with aquatic invertebrates. We fit our model using JAGS via the “rjags” package (Plummer 2015). We used trophic enrichment factors of 0.4‰ ± 1.3‰ for δ^13^C and 3.4‰ ± 1.0‰ for δ^15^N (Post 2002). For each site, we used “uninformative” priors and a predefined “long” parameter set of Markov Chain Monte Carlo (MCMC) simulation to estimate mean dietary contributions of prey to predators, consisting of three chains of 300,000 iterations, a burn‐in phase of 200,000, and thinned by a factor of 100. Numerical diagnostics were evaluated based on a $\hat{R}$ ≤ 1.01 (Vehtari et al. 2021). All statistical analyses were performed in R version 4.2.0 (R Core Team 2022).

## 4 Results

### 4.1 Isotopic Composition and Niche Overlaps

We collected 302 individual fishes comprising 18 species (Table 1) and 42 aquatic invertebrate samples at all sites (see Table S1 for a full list). In addition, isospace biplots of all taxa per site, including prey items, are included in the Figures S1–S5. See also Rojas Carbajal (2023) for a visual representation of the isotopic values of all taxa (convex hulls biplots).

**TABLE 1:** List and distribution of fish species collected during 2014 and 2015 summer sampling of Green Bay coastal wetlands. Only species with n≥3 were used to calculate stable isotope community metrics.

Common name	Species	Species code	Feeding guild	Number of counts per site				
				CEDA [R]	LIST [L]	PENS [R]	PESH [R]	PTSA [L]
Bluegill	*Lepomis macrochirus*	BLG	Invertivore	1	4	6		3
Bluntnose minnow	*Pimephales notatus*	BNM	Benthivore			1	3	
Bowfin	*Amia calva*	BON	Piscivore	1	5	11	14	6
		BON.YOY	Planktivore			12	2	
Burbot	*Lota lota*	BUT	Piscivore					3
Common shinner	*Luxilus cornutus*	COS	Invertivore	3		3	3	
Gizzard shad	*Dorosoma cepedianum*	GIS	Planktivore			1		3
Green sunfish	*Lepomis cyanellus*	GSF	Invertivore			3		
Largemouth bass	*Micropterus salmoides*	LMB	Piscivore		2	3	1	3
		LMB.YOY	Invertivore		8	11	6	4
Northern pike	*Esox lucius*	NOP	Piscivore	4	3	2	8	3
		NOP.YOY	Piscivore		4	3	6	2
Pumpkinseed	*Lepomis gibbosus*	PUS	Invertivore	2	8	6	6	
Rock bass	*Ambloplites rupestris*	ROB	Invertivore	5	3	6	6	1
Round goby	*Neogobius melanostomus*	ROG	Invertivore		3	1		
Smallmouth bass	*Micropterus dolomieu*	SMB	Piscivore	12		4	2	4
		SMB.YOY	Invertivore				3	1
Spotfin shiner	*Cyprinella spiloptera*	SFS	Invertivore				3	
Walleye	*Sander vitreus*	WAE	Piscivore		2	5	1	3
		WAE.YOY	Planktivore				5	
White sucker	*Catostomus commersoni*	WHS	Benthivore	3				
Yellow perch	*Perca flavescens*	YEP	Piscivore	3	4	6	5	6
		YEP.YOY	Benthivore		6	6	7	3

Abbreviations: CEDA, Cedar River; LIST, Little Sturgeon Bay; PENS, Pensaukee River; PESH, Peshtigo River; PTSA, Point au Sable; Wetland ecotype: [R] riverine, [L] lacustrine.

Overall, samples from Cedar River and Little Sturgeon Bay showed the broadest ranges in δ^13^C isotope values (Cedar River: −34.93‰ to −17.82‰; Little Sturgeon Bay: −38.02‰ to −18.72‰), while Peshtigo River showed the broadest range in δ^15^N values (3.63‰–15.39‰). Although the Point au Sable samples had a narrow range of δ^15^N values, all feeding guilds occupied higher trophic levels than at other sites.

We found a high probability of more than 70% of piscivorous fish overlapping in the invertivores isotopic niche in Cedar River, Little Sturgeon Bay, and Peshtigo River. Likewise, the pairwise niche overlap between piscivores and benthivores was high in Peshtigo River (> 75%) (Figure 2). Niche overlap probabilities are displayed in the Figures S1–S10.

**FIGURE 2:**
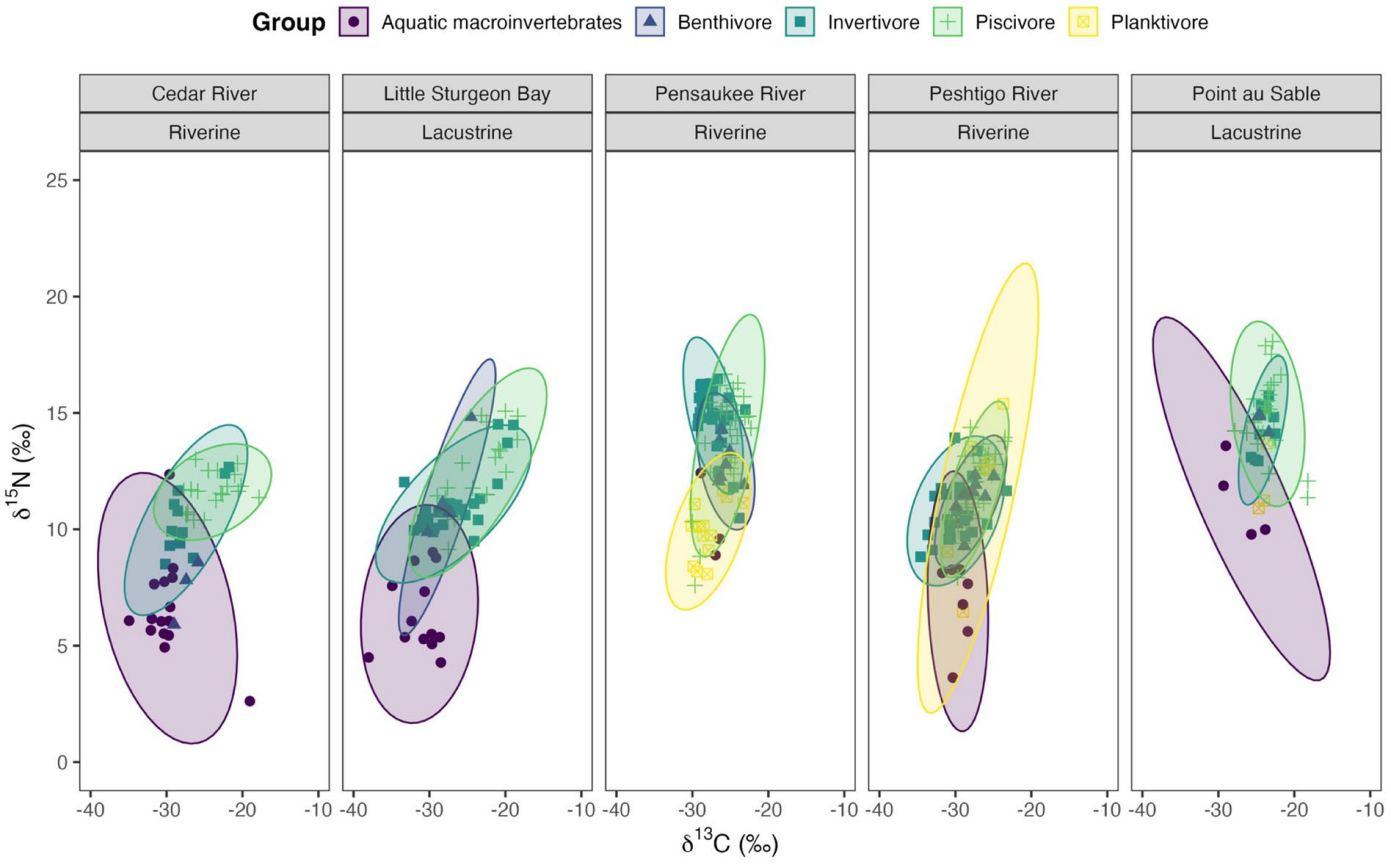
δ^13^C and δ^15^N biplots of feeding guilds for each Green Bay wetland site. The standard ellipses area (SEA) represents 95% of the bivariate distribution of aquatic communities at each wetland site.

In Figure 2, we observe that benthivorous, invertivorous, and piscivorous fish had the lowest mean δ^13^C and δ^15^N values at Peshtigo River and Cedar River, respectively. Planktivorous fish had the smallest mean δ^13^C and δ^15^N values at Pensaukee River. Furthermore, all feeding guilds had the greatest mean δ^13^C and δ^15^N values at Point au Sable except for invertivorous fish, which had the greatest mean δ^15^N value at Pensaukee River (Table 2). Moreover, aquatic invertebrates had the lowest mean δ^13^C and δ^15^N values at Little Sturgeon Bay and the highest mean δ^13^C and δ^15^N values at Point au Sable.

**TABLE 2:** Raw mean (SD) values of δ^13^C and δ^15^N (in ‰) for aquatic invertebrates, fish species grouped by feeding guilds, and top fish predators collected at each site. Site codes are indicated in Table 1.

Group	CEDA [R]		LIST [L]		PENS [R]		PESH [R]		PTSA [L]	
	δ^13^C (SD)	δ^15^N (SD)	δ^13^C (SD)	δ^15^N (SD)	δ^13^C (SD)	δ^15^N (SD)	δ^13^C (SD)	δ^15^N (SD)	δ^13^C (SD)	δ^15^N (SD)
Aquatic invertebrates	−29.87 (3.36)	6.61 (2.13)	−31.32 (2.77)	6.37 (1.68)	−27.42 (1.31)	10.30 (1.87)	−29.67 (1.24)	6.91 (1.74)	−26.95 (2.67)	11.31 (1.79)
Benthivore	−27.42 (1.59)	7.44 (1.37)	−27.96 (2.01)	11.38 (1.75)	−25.85 (1.23)	12.92 (0.92)	−28.16 (1.58)	11.19 (0.98)	−24.61 (0.75)	14.65 (0.42)
Invertivore	−27.53 (2.71)	10.40 (1.73)	−27.10 (3.91)	11.09 (1.30)	−27.49 (1.60)	14.88 (1.32)	−30.17 (2.59)	10.79 (1.24)	−24.50 (1.06)	14.25 (1.07)
Piscivore	−24.09 (3.03)	11.60 (0.78)	−23.91 (3.37)	12.43 (1.70)	−25.55 (1.90)	14.02 (2.03)	−27.61 (1.73)	11.71 (1.49)	−23.80 (1.80)	14.96 (1.54)
Planktivore					−28.04 (1.99)	9.92 (1.21)	−27.27 (2.60)	11.77 (3.01)	−24.08 (0.56)	11.94 (1.52)
Top predators										
Bowfin	−26.66 (0.00)	10.40 (0.00)	−21.75 (2.79)	12.82 (0.92)	−25.89 (1.53)	12.98 (2.09)	−28.21 (1.20)	10.86 (1.63)	−23.49 (3.91)	13.43 (1.36)
Largemouth bass			−19.57 (1.20)	14.97 (0.15)	−24.99 (0.87)	16.22 (0.48)	−28.37 (0.00)	14.37 (0.00)	−23.68 (0.58)	17.82 (0.28)
Northern pike	−22.93 (1.88)	11.51 (0.87)	−24.53 (4.15)	12.01 (0.93)	−24.62 (0.75)	14.39 (0.52)	−25.69 (1.56)	12.83 (0.89)	−23.82 (0.28)	15.35 (0.40)
Smallmouth bass	−23.36 (3.07)	11.96 (0.60)			−24.40 (1.88)	15.35 (0.91)	−26.67 (2.11)	12.78 (0.60)	−23.45 (1.32)	15.70 (1.37)
Walleye			−23.17 (0.47)	14.75 (0.19)	−23.69 (1.10)	15.08 (0.43)	−29.08 (0.00)	13.14 (0.00)	−23.68 (0.30)	14.62 (1.95)
Yellow perch	−27.68 (0.27)	10.67 (0.06)	−25.20 (3.10)	11.21 (1.79)	−27.48 (1.65)	14.13 (1.97)	−28.57 (1.42)	11.59 (1.00)	−24.69 (0.93)	14.90 (0.50)

Abbreviations: CEDA, Cedar River; LIST, Little Sturgeon Bay; PENS, Pensaukee River; PESH, Peshtigo River; PTSA, Point au Sable; Wetland ecotype: [R] riverine, [L] lacustrine.

Mean δ^13^C values were lowest for all top predator species at Peshtigo River. Mean δ^13^C values were highest at Little Sturgeon Bay for bowfin, largemouth bass, and walleye; for northern pike and smallmouth bass in Cedar River; and for yellow perch in Point au Sable. In addition, the lowest mean δ^15^N values were observed for bowfin, northern pike, smallmouth bass, and yellow perch at Cedar River and for largemouth bass and walleye in Peshtigo River. The highest mean δ^15^N values for all species occurred at Point au Sable, except for walleye (highest mean δ^15^N value at Pensaukee River, Table 2).

### 4.2 Trophic Position of Top Predator Fish Species

We found significant differences in the mean trophic positions of bowfin, largemouth bass, northern pike, and walleye among sites (one‐way ANOVA, p<0.05), but not for smallmouth bass and yellow perch (p>0.10) (Table 3). We observed significant differences in the trophic position of bowfin and largemouth bass (1) between the Pensaukee River and Little Sturgeon Bay and (2) between Point au Sable and Little Sturgeon Bay (TukeyHSD, p<0.05). In addition, the trophic position of northern pike did not differ significantly between the Peshtigo River and the Pensaukee River (TukeyHSD, p>0.05).

**TABLE 3:** Mean values of trophic position (standard deviation) for top predators at each site after correcting isotopic baselines. Outputs of the one‐way ANOVA (analysis of variance) of the significant differences in the trophic position (TP) of the same top predator species among sites. Statistically significant differences at *p* < 0.05 are indicated in bold. Site codes are indicated in Table 1.

Species	TP (SD)					df	*F*	*p*
	CEDA [R]	LIST [L]	PENS [R]	PESH [R]	PTSA [L]			
Bowfin	3.16 (0.00)	4.11 (0.27)	2.79 (0.61)	3.16 (0.48)	2.85 (0.40)	4	6.742	**0.001**
Largemouth bass		4.75 (0.04)	3.74 (0.14)	4.20 (0.00)	4.14 (0.08)	3	37.02	**0.001**
Northern pike	3.49 (0.26)	3.88 (0.27)	3.20 (0.15)	3.74 (0.26)	3.41 (0.12)	4	3.675	**0.028**
Smallmouth bass	3.62 (0.17)		3.48 (0.27)	3.73 (0.18)	3.52 (0.40)	3	0.676	0.578
Walleye		4.68 (0.06)	3.41 (0.13)	3.83 (0.00)	3.20 (0.57)	3	9.885	**0.006**
Yellow perch	3.24 (0.02)	3.64 (0.53)	3.13 (0.58)	3.38 (0.30)	3.28 (0.15)	4	1.094	0.388

Abbreviations: CEDA, Cedar River; LIST, Little Sturgeon Bay; PENS, Pensaukee River; PESH, Peshtigo River; PTSA, Point au Sable; Wetland ecotype: [R] riverine, [L] lacustrine.

**FIGURE 3:**
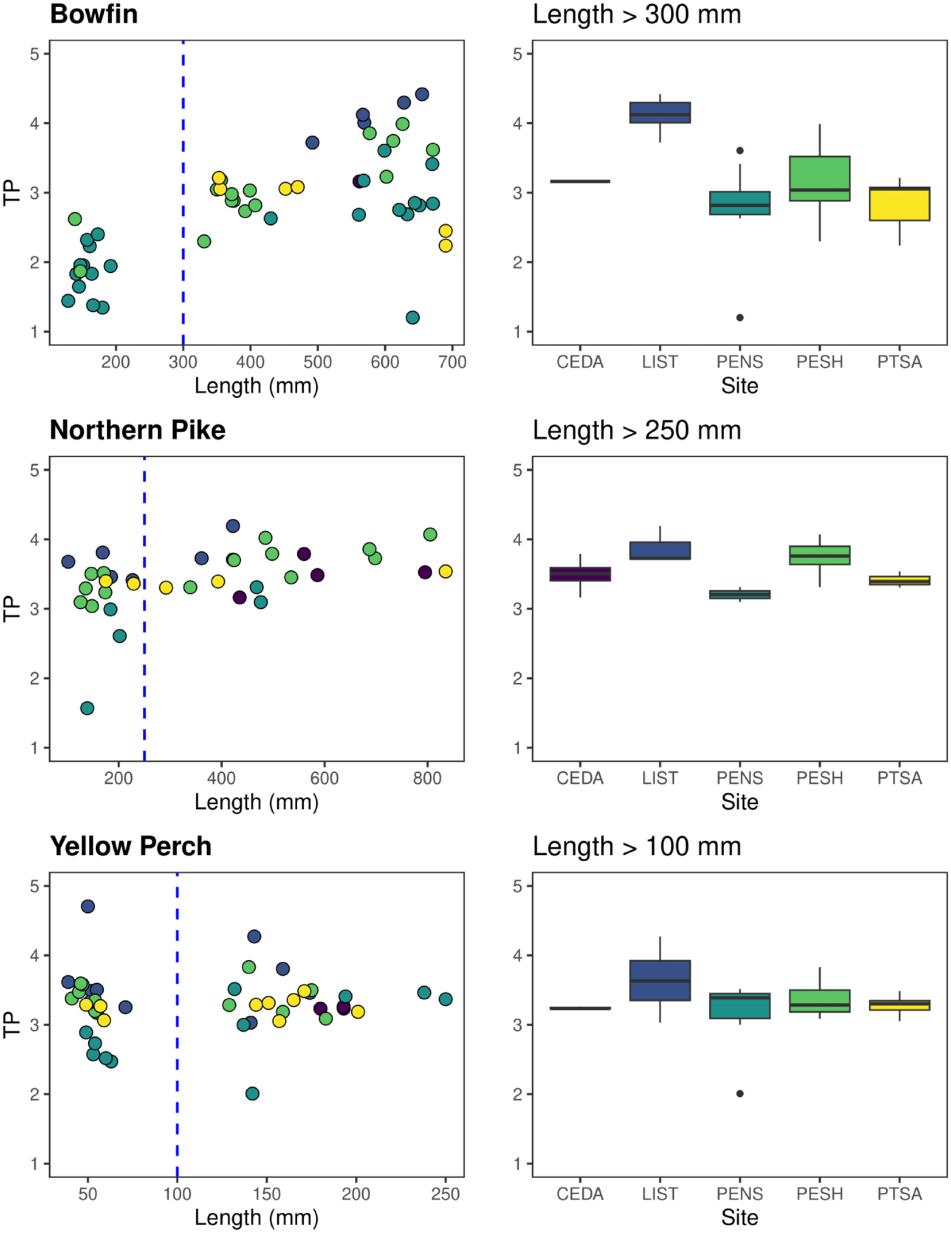
Trophic position compared to total length (mm) of bowfin *Amia calva* , northern pike *Esox lucius* , and yellow perch *Perca flavescens* in all Green Bay sites. Right figures indicate trophic positions for fish larger than the length threshold (dashed blue line), above which individuals are predominantly piscivorous. CEDA, Cedar River; LIST, Little Sturgeon Bay; PENS, Pensaukee River; PESH, Peshtigo River; PTSA, Point au Sable.

Bowfin and northern pike occupied different trophic positions at all sites after shifting their diet to primarily piscivorous (length threshold greater than 300 and 250 mm, respectively; Figure 3). For both species, we observed an increase in the trophic position with size. Despite changes in size and diet, yellow perch presented similar isotopic values, and no significant differences were found among sites.

### 4.3 Trophic Structure and Isotopic Niche of Piscivorous Community

**FIGURE 4:**
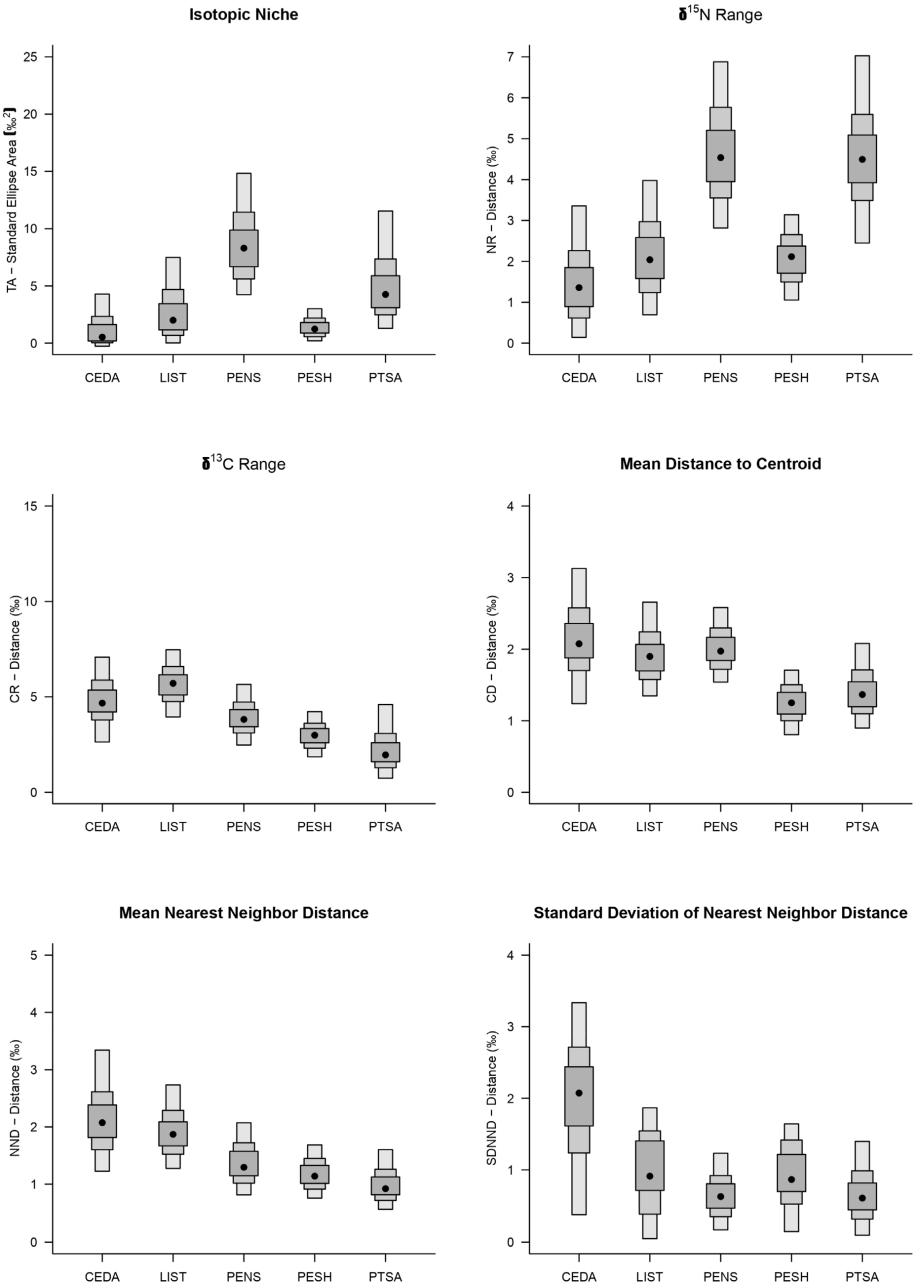
Results for Bayesian community isotope metrics that provide information on isotopic niche area, trophic diversity (δ^15^N range, δ^13^C range, mean distance to centroid), and trophic redundancy (mean nearest neighbor distance and standard deviation of nearest neighbor distance) of piscivorous fish in Green Bay coastal wetlands. Black circles indicate the mode of the SEA with Bayesian credible intervals (50%, 75%, and 95%, from dark to light) indicated by the boxes. Site codes are indicated in Table 1. CEDA, Cedar River; LIST, Little Sturgeon Bay; PENS, Pensaukee River; PESH, Peshtigo River; PTSA, Point au Sable.

Isotope‐based community metrics suggested differences in the trophic structure of piscivorous fish communities, that is, those comprising top predators and YOY northern pike, among sites (Figure 4). The piscivorous fish community at Pensaukee River had the largest isotopic niche area (TA) and Cedar River the smallest. Pensaukee River also had the highest median NR value, indicating more trophic levels than other sites, whereas the Cedar River had the lowest median NR value. Little Sturgeon Bay had the highest median CR value (i.e., high diversity of basal resources) and Point au Sable had the lowest (i.e., lower diversity of basal resources). Cedar River had the highest mean CD value (wider isotopic niche) and Peshtigo the lowest (narrow isotopic niche) compared to other sites. The highest NND and SDNND values were observed at Cedar River, indicating a lower and uneven proportion of species of trophic niches (i.e., lower trophic redundancy), in contrast to Point au Sable and Pensaukee River, which had the lowest NND and SDNND values, respectively.

**FIGURE 5:**
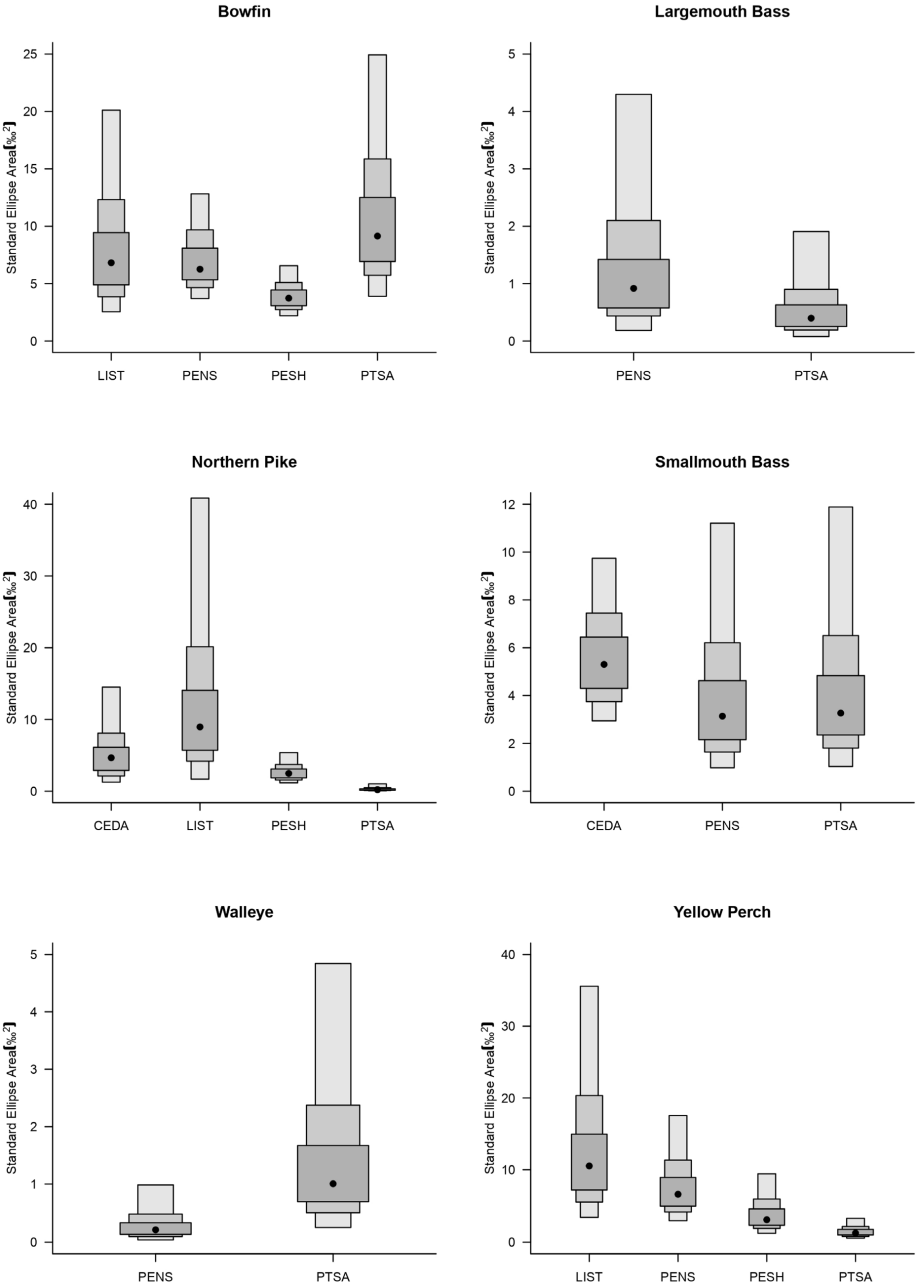
Isotopic niche area of predatory fish in Green Bay coastal wetlands. Black circles indicate the mode of the SEA with Bayesian credible intervals (50%, 75%, and 95%, from dark to light) indicated by the boxes. Site codes are indicated in Table 1. CEDA, Cedar River; LIST, Little Sturgeon Bay; PENS, Pensaukee River; PESH, Peshtigo River; PTSA, Point au Sable.

**FIGURE 6:**
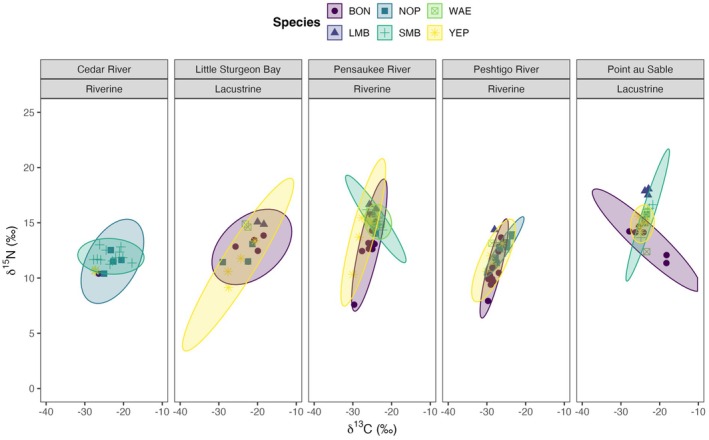
δ^13^C and δ^15^N biplots of top predator fish species of each wetland site in Green Bay. The standard ellipses area (SEA) represents 95% of the bivariate distribution of predator communities at each wetland site. Species and site codes are indicated in Table 1.

We also found that the isotopic niches (represented by SEA_B_) of the top predators differed among sites (Figure 5) and the interspecific overlap varied among species (Figure 6). The northern pike and yellow perch had higher isotopic niche values at Little Sturgeon Bay, and bowfin and walleye had higher values at Point au Sable. The largemouth bass and smallmouth bass had the largest isotopic niches in the Pensaukee River and Cedar River, respectively. At Point au Sable, the isotopic niche of bowfin was larger than that of other predators, while the niche of northern pike was the smallest (Figure 5). The niches for northern pike and smallmouth bass are partially overlapped, thus specialized on the same diet (i.e., redundant), at Cedar River but were nested at Point au Sable (Figure 6). In addition, the niches of bowfin and northern pike are partially overlapped in Little Sturgeon Bay and Peshtigo River. The yellow perch niche was relatively narrow at most sites and showed little variation in breadth except at Point au Sable.

### 4.4 Resource Contributions to Top Predator Fish Communities

**FIGURE 7:**
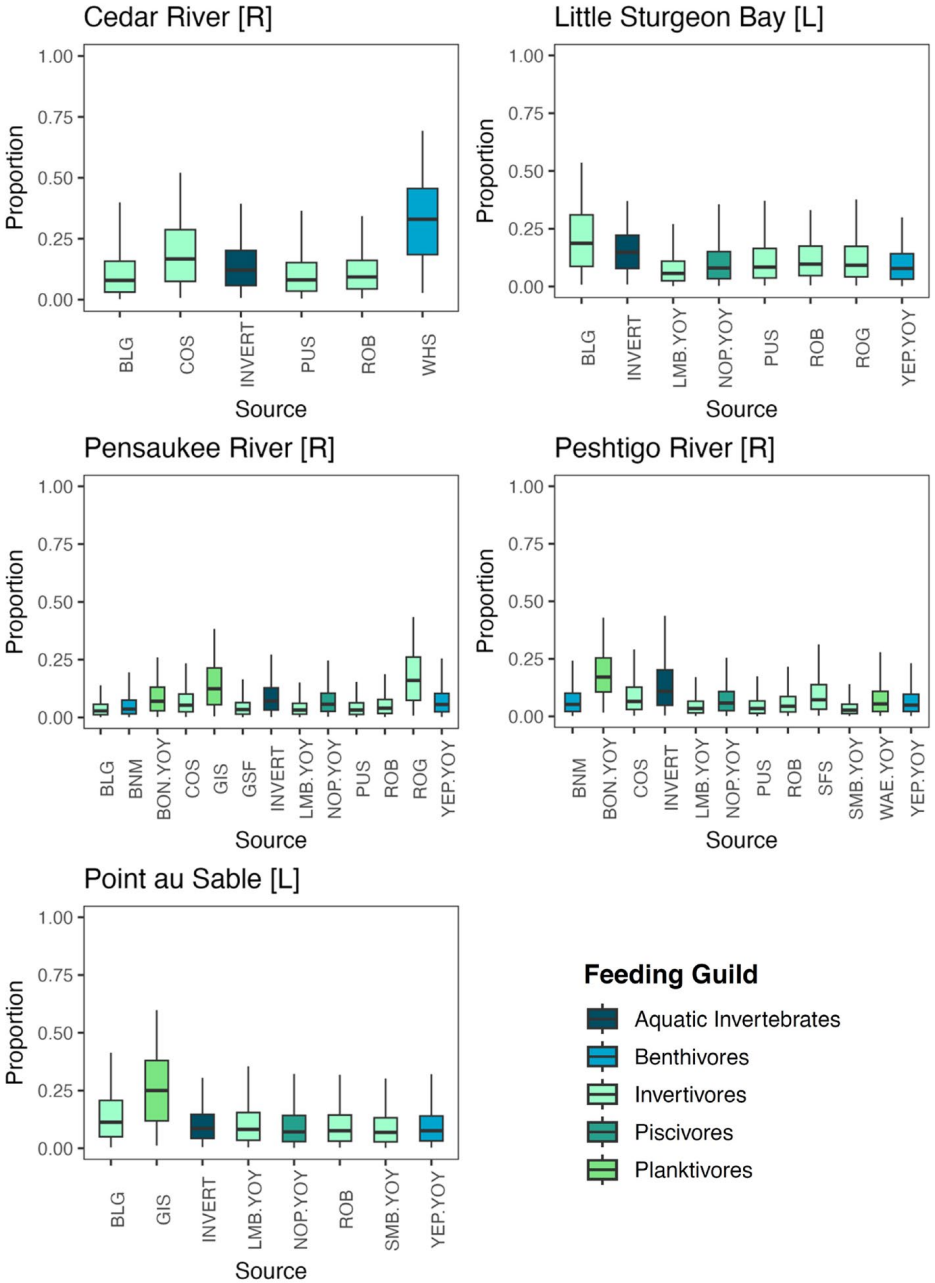
Bayesian mixing model estimates of overall predatory fish community diet proportions from various feeding guilds at each wetland site. Higher percent contributions indicate a larger proportion of diet is comprised of that source (i.e., prey). The horizontal black line represents the median posterior estimate, the box represents the upper and lower quartiles, and the vertical line represents the 95% credible interval. Species codes are indicated in Table 1. L, lacustrine; R, riverine.

Results of the mixing model for top predator fish species suggested that their diet resources consisted primarily of forage fish in riverine wetlands (Cedar River, Pensaukee River and Peshtigo River). Conversely, in lacustrine wetlands (Little Sturgeon Bay and Point au Sable) top predators diversified their diet resources between aquatic invertebrates and forage fish (Figure 7). While our mixing model provides insights into potential dietary contributions, we recognize the limitations of underdetermined systems. Our model may struggle to differentiate among sources, leading to potential artifacts in the estimated contributions of different end members. Therefore, these results should be considered as approximations constrained by the model's assumptions and not as direct evidence of generalized diets or equal foraging.

**FIGURE 8:**
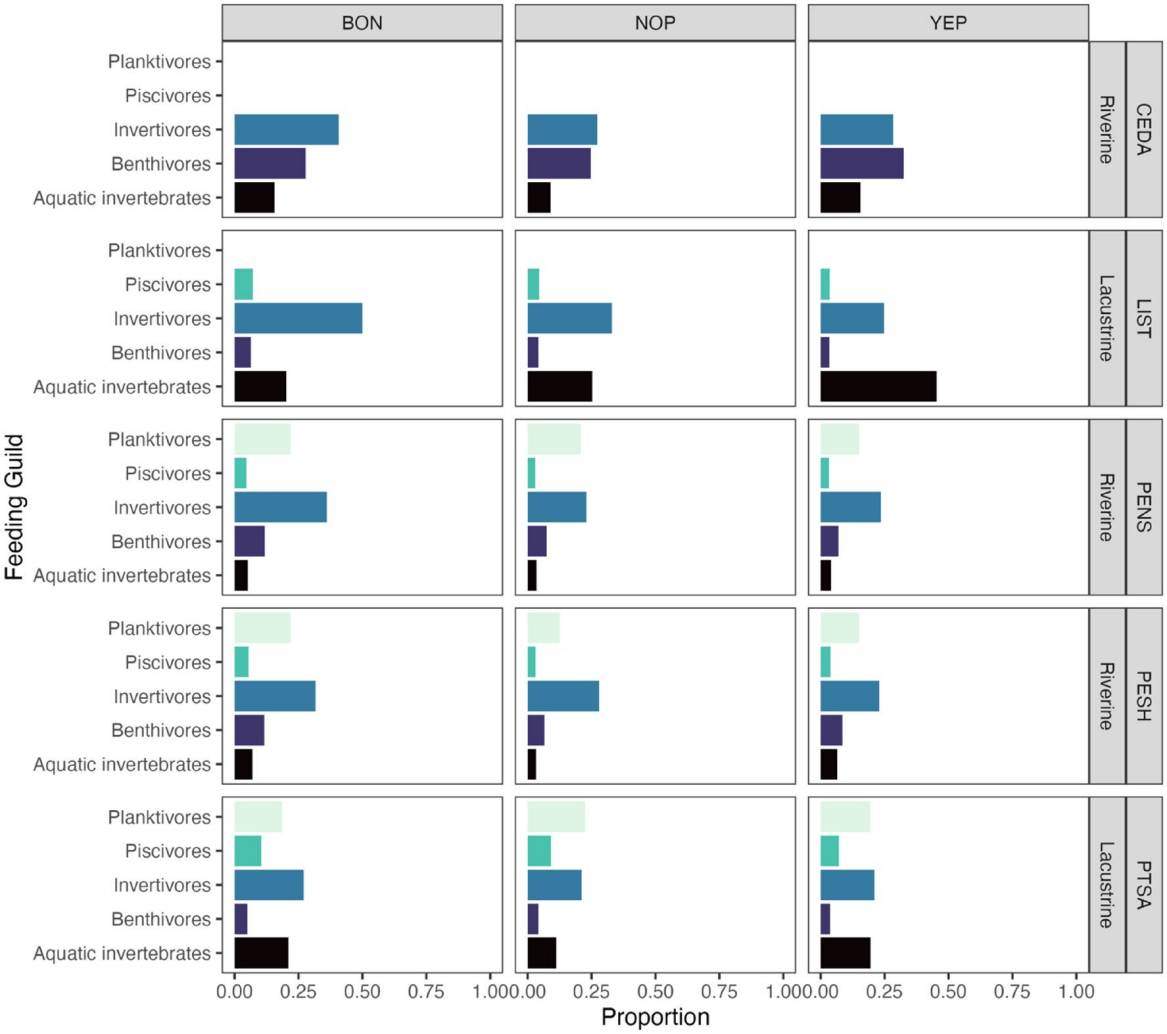
Proportions in diet based on median posterior estimates of bowfin *Amia calva* (BON), northern pike *Esox lucius* (NOP), and yellow perch *Perca flavescens* (YEP) in all Green Bay sites. Species and site codes are indicated in Table 1. CEDA, Cedar River; LIST, Little Sturgeon Bay, PENS, Pensaukee River; PESH, Peshtigo River; PTSA, Point au Sable.

The bowfin diet resources comprised more than 30% invertivorous fish in all sites, but about 20% planktivorous fish in the Pensaukee River, Peshtigo River, and Point au Sable (Figure 8). Diet proportions for northern pike indicate more than 20% of invertivorous and planktivorous fish each in all sites where they were sampled. Conversely, the diet resources of yellow perch consisted of more than 30% benthivorous and invertivorous fish each in Cedar River; 45% aquatic invertebrates and 25% invertivorous fish in Little Sturgeon Bay; over 23% invertivorous fish in Pensaukee River and Peshtigo River; and up to 20% invertivorous fish and aquatic invertebrates each in Point au Sable. Diet contributions per source are provided in the Figures S11–S16, for each top predator.

## 5 Discussion

From 1989 to 2019, Green Bay in Lake Michigan experienced a decadal decline in angler sport harvest of fish species (northern pike, smallmouth bass, walleye, and yellow perch), but the causes remain unresolved. We sought to gain insight into this decline by examining habitat preferences, trophic position, and dietary plasticity of these putative piscivores. We hypothesized that coastal wetlands are important for the foraging and rearing of these species, as these wetlands should influence the energetic costs of feeding and the energy return of consumed prey tissue within the Green Bay aquatic system while improving its resilience.

### 5.1 Interspecific Isotopic Niche Overlap Among Fish Communities and Diet Contributions to Top Predators

We found a high overlap among the isotopic niches of piscivorous, invertivorous, and benthivorous fish, reflecting the capacity of different feeding guilds to exploit isotopically similar sources. Invertivorous fish contributed a large amount to the top predator's diet resources, ranging from 11% to 58% across all sites.

Our results suggest that predatory fish communities in Green Bay coastal wetlands displayed different trophic features and energy sources, resulting in varying isotopic values in the food web. We observed differences in δ^13^C and δ^15^N values among sites, as well as more enriched composition for certain feeding guilds, such as in fish invertivores or planktivores, which, therefore, occupied higher trophic levels. Piscivorous fish generally occupy higher trophic levels than other feeding guilds. However, in riverine wetlands (Pensaukee River and Peshtigo River) invertivores, piscivores, and planktivores were isotopically similar.

Moreover, the piscivorous fish communities in Green Bay had different trophic features at each site. The piscivore community at Point au Sable had the smallest TA and, therefore, a more specialized trophic niche compared to other sites or, alternatively, the least inherent variability in its isotopic biochemistry. The latter should be further explored in future studies. The NR of the piscivore community in the Pensaukee River and Point au Sable was higher than at other sites, indicating that these sites had more trophic levels. The CR was higher in Little Sturgeon Bay, indicating a broader utilization of isotopically distinct basal resources. The trophic diversity in Little Sturgeon Bay and Pensaukee River was also explained by the greater niche width and interspecies spacing (i.e., higher CD values). Conversely, Point au Sable had smaller NND, SDNND, and CR values which suggested a nonlinear reduction in trophic diversity, although the piscivore community may have had a higher trophic redundancy (Burdon et al. 2020).

Due to the direct connection between the watershed and the lake, coastal wetlands are often sites of high primary productivity. Tributaries deliver large nutrient loads (e.g., phosphorus, nitrogen) into coastal wetlands (Mooney et al. 2020) that can drive algal production (Cooper et al. 2016). Because community‐level δ^13^C values and isotopic niches of feeding guilds (whose foraging habits depend mainly on primary production levels) are responsive to changes in habitat conditions (e.g., water quality altered by nitrogen pollution) (Wang et al. 2021), changes in niche breadths can lead to changes in fish assemblages (Zorn and Kramer 2022).

### 5.2 Variation of Trophic Position of Top Predators

We found differences in isotopic values that may reflect variability in habitat and resource utilization but could also result from various environmental and anthropogenic factors. Most of the top predators in this study showed differences in the trophic position they occupied across sites, except for smallmouth bass and yellow perch. Green Bay is composed of heterogeneous and dynamic habitats (Brazner and Beals 1997) leading to diverse fish communities with different trophic ecologies. For instance, bowfin and northern pike presented significant differences in the mean trophic position across all sites. Nevertheless, the rise in nitrogen inputs from agriculture and urban development in the surrounding watersheds (Han and Allan 2012; Mooney et al. 2020) would lead to biological fixation and higher isotopic values downstream.

Trophic position of fishes generally increases with increasing total length as gape size increases and fish undergo ontogenetic diet shifts (Murphy et al. 2019), but variation in baseline δ^15^N values (habitat‐specific characteristics) determines the type of resources they consume (Vander Zanden and Rasmussen 1999) or could cause shifts in δ^15^N independently of a change in diet. Ontogenetic diet shifts may also influence the availability of resources for other predatory fishes, such as YOY largemouth bass and yellow perch.

Our results suggest that top predators adjust their isotopic niches based on the habitat they feed in, switching their diet and supporting the findings of Flaherty and Ben‐David (2010). This plasticity suggests that the top predators are diversifying their diet, niche space, and trophic position in response to conditions at each site, revealing resource partitioning. These changes may occur due to the availability of more diverse prey resources and/or from isotopically distinct habitats (Newsome et al. 2007) due to changes in spawning, nursery, and foraging habitats. For instance, we found that yellow perch and other top predators may be competing for similar resources, as evidenced by the high overlap of the isotopic niche. Although yellow perch exploit both coastal and nearshore habitats (Sierszen et al. 2019), this species is consistently a wetland‐dependent species (more abundant in wetland habitats); thus, its foraging behavior could be associated more strictly with wetland‐type habitats compared to other species that more actively or partially migrate. However, yellow perch migration patterns are still unclear, especially in Green Bay.

### 5.3 Influence of Wetland‐Habitat Ecotypes on Dietary Resources

We found that top predators diversified their diet in lacustrine wetlands but had a distinct foraging habitat preference in riverine wetlands, emphasizing the importance of habitat type and structure in feeding diversity. We observed that the hydrogeomorphology of coastal wetlands in Green Bay played a key role in the trophic structure and isotopic niches of the biota, ultimately driving the energy support for the system. In general, lacustrine wetlands, such as Little Sturgeon Bay, hosted generalist top predators with the highest trophic position due to enriched prey resources (i.e., higher δ^15^N values). Conversely, riverine wetlands often had specialist predators with broader isotopic niches, which offered more stability to the system.

Results of mixed models showed variations in the diet of the main predators between Green Bay sites. In lacustrine wetlands such as Little Sturgeon and Point au Sable, top predators diversified their diet between aquatic invertebrates and forage fish, while in riverine wetlands, they primarily consumed forage fish. Forage fish that contributed primarily to the predator diet included invertivorous fishes, such as bluegills, and prey that was isotopically similar. Top predators used multiple isotopically distinct basal resources or a high niche diversification at lower trophic levels at Pensaukee River, as evidenced by the large isotopic niche of the community.

We acknowledge that the isotopic gradient within a group could not reflect feeding differences but habitat differences owing to Lake Michigan sources in the diet. When estimating the trophic position for migratory fishes, such as northern pike, we assumed a single source (wetland prey) and not a dual source (lake and wetland prey). Therefore, our interpretation of the isotopic data assumes that the wetland where fish were caught serves as their primary habitat. Our assumption is based on preliminary data (unpublished) that showed that wetland‐caught samples exhibited similar isotopic signatures to nearshore‐caught samples, although migratory fish may mix isotopically as they pass through different habitats. In addition, we would like to mention that sample sizes for these two species (walleye and northern pike) are relatively low while collecting them from the lake can be challenging. Nevertheless, it is important to acknowledge that many fish species in this study are migratory, routinely moving between the open waters of the lake and coastal wetlands. This migratory behavior introduces complexity to isotopic studies and requires a sound approach when handling and interpreting data for migratory species compared to resident species. We suggest that future studies should be addressed to account for these dual sources and incorporate tracking or movement data to refine our understanding of habitat and resource use across these systems.

The effects of habitats ecotype in the shape and size of isotopic niches of top predator communities could be also a population level phenomenon that can be explored in future studies at broader scales. Variations in dietary overlap and niche mechanisms, along with environmental conditions in coastal wetlands, underpin the ability of fish communities in Green Bay to respond to different stressors that are apparent in this region; thus, further research needs to address these responses. Furthermore, how habitat degradation may impact Green Bay food webs (wetland and nearshore systems) should be included in the agenda to promote fisheries management in Lake Michigan.

## Author Contributions

**Tania V. Rojas:** data curation (lead), formal analysis (lead), investigation (equal), visualization (equal), writing – original draft (lead), writing – review and editing (equal). **Katherine E. O'Reilly:** methodology (equal), supervision (equal), validation (equal), writing – original draft (lead), writing – review and editing (equal). **Christopher J. Houghton:** conceptualization (equal), investigation (equal), methodology (equal), project administration (equal), resources (equal), supervision (equal), validation (equal), writing – original draft (lead), writing – review and editing (equal). **Jeremiah S. Shrovnal:** data curation (equal), investigation (equal), methodology (equal), validation (equal), writing – review and editing (equal). **Martin B. Berg:** conceptualization (equal), funding acquisition (equal), investigation (equal), methodology (equal), resources (equal), validation (equal), writing – review and editing (equal). **Donald G. Uzarski:** conceptualization (equal), funding acquisition (equal), investigation (equal), methodology (equal), resources (equal), validation (equal), writing – review and editing (equal). **Gary A. Lamberti:** conceptualization (equal), funding acquisition (equal), investigation (equal), methodology (equal), resources (equal), validation (equal), writing – review and editing (equal). **Patrick S. Forsythe:** conceptualization (equal), funding acquisition (equal), investigation (equal), methodology (equal), resources (equal), supervision (equal), validation (equal), writing – original draft (lead), writing – review and editing (equal).

## Conflicts of Interest

The authors declare no conflicts of interest.

## Supporting information


Data S1.

## Data Availability

The data is available at https://github.com/taniarojasc/GreenBayCoastalWetlandsFoodWebs.git.
