# Supplementary material for: Coastal Wetlands Drive Isotopic Niche Plasticity of Top Predator Fish Communities in Green Bay, Lake Michigan (USA)
Source: Ecol Evol. 2025 May 23;15(5):e71463. doi: 10.1002/ece3.71463 (PMC12102075; doi:10.1002/ece3.71463)
Supplement: Supplementary file 1 — Data S1. [file ECE3-15-e71463-s001.docx]

Coastal Wetlands Drive Isotopic Niche Plasticity of Top Predator Fish Communities in Green Bay, Lake Michigan (USA)

Tania V. Rojas^* 1,2^, Katherine E. O’Reilly ^3^, Christopher J. Houghton ^1^, Jeremiah S. Shrovnal ^4^, Martin B. Berg ^5^, Donald G. Uzarski ^6^, Gary A. Lamberti ^7^, Patrick S. Forsythe ^1^

^1^ Aquatic Ecology and Fisheries Laboratory, Department of Natural and Applied Sciences, University of Wisconsin-Green Bay, Green Bay, Wisconsin, USA

^2^ Department of Biology, University of Kentucky, Lexington, Kentucky, USA

^3^ Illinois-Indiana Sea Grant, Champaign, Illinois, USA

^4^ Wisconsin Department of Natural Resources, Bureau of Fisheries Management, Bayfield, Wisconsin, USA

^5^ Department of Biology, Loyola University Chicago, Chicago, Illinois, USA

^6^ Institute for Great Lakes Research, Central Michigan University, Mount Pleasant, Michigan, USA

^7^ Department of Biological Sciences, University of Notre Dame, Notre Dame, Indiana, USA

^*^ Corresponding author: tvrojas02@gmail.com

# Supplementary Material

Supplemental Table 1 List of aquatic invertebrates collected during 2014 and 2015 summer sampling of Green Bay coastal wetlands. CEDA: Cedar River; LIST: Little Sturgeon Bay, PENS: Pensaukee River; PESH: Peshtigo River; PTSA: Point au Sable. Wetland Ecotype: [R] Riverine, [L] Lacustrine

| Order/Species | Number of counts per site | | | | |
| --- | --- | --- | --- | --- | --- |
|  | CEDA [R] | LIST [L] | PENS [R] | PESH [R] | PTSA [L] |
| Amphipod | 4 | 3 |  |  |  |
| Belostomatidae |  |  |  | 1 |  |
| Chironomidae | 3 | 1 | 1 |  |  |
| Coleoptera |  |  | 1 | 1 |  |
| Ephemeroptera |  | 1 |  |  |  |
| Gastropoda | 2 |  |  |  | 3 |
| Gyrinidae |  |  |  | 1 |  |
| Hemiptera | 1 | 2 | 1 | 1 |  |
| Isopod | 2 | 3 |  | 2 |  |
| Notonectidae |  |  |  | 1 |  |
| Odonata | 2 | 3 |  |  | 1 |
| Trichoptera | 1 |  |  |  |  |


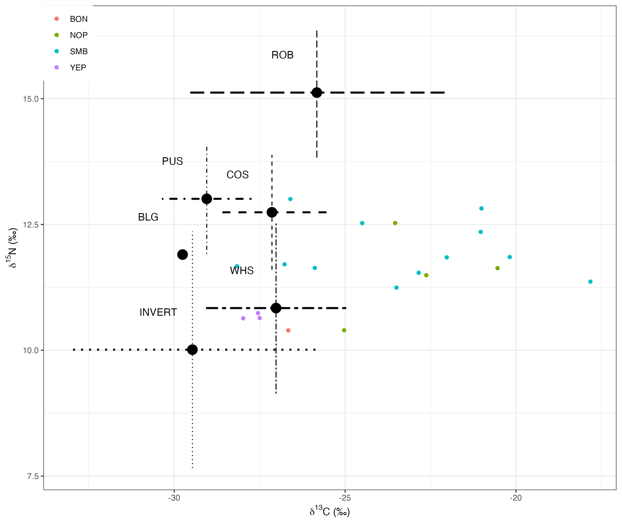

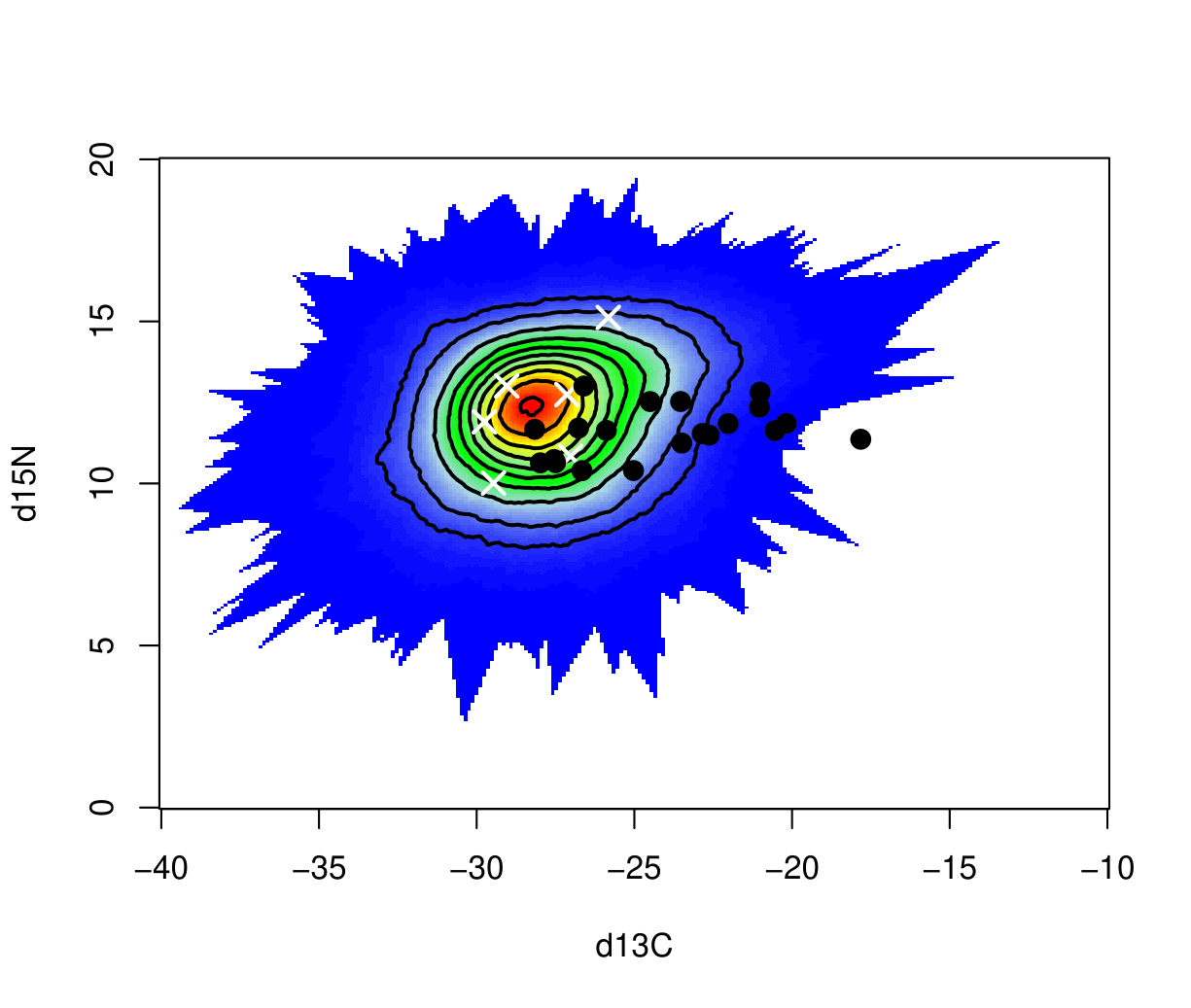


Supplemental Figure 1 Left: Raw isospace plot of fish species in Cedar River (riverine wetland). Species codes are indicated in Table 1. Right: The simulated mixing region for the biplot shown on the left.


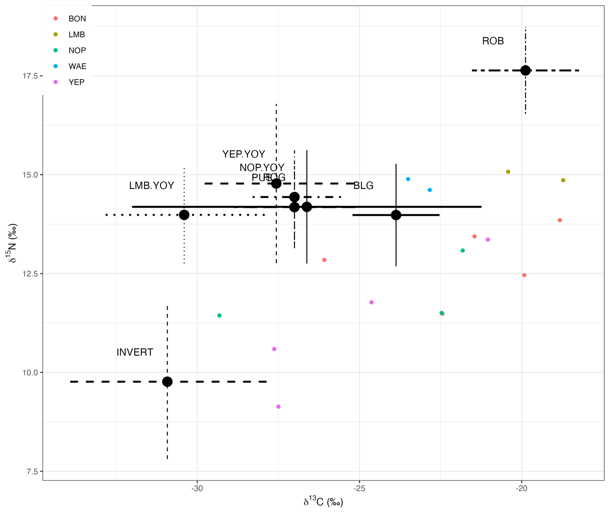

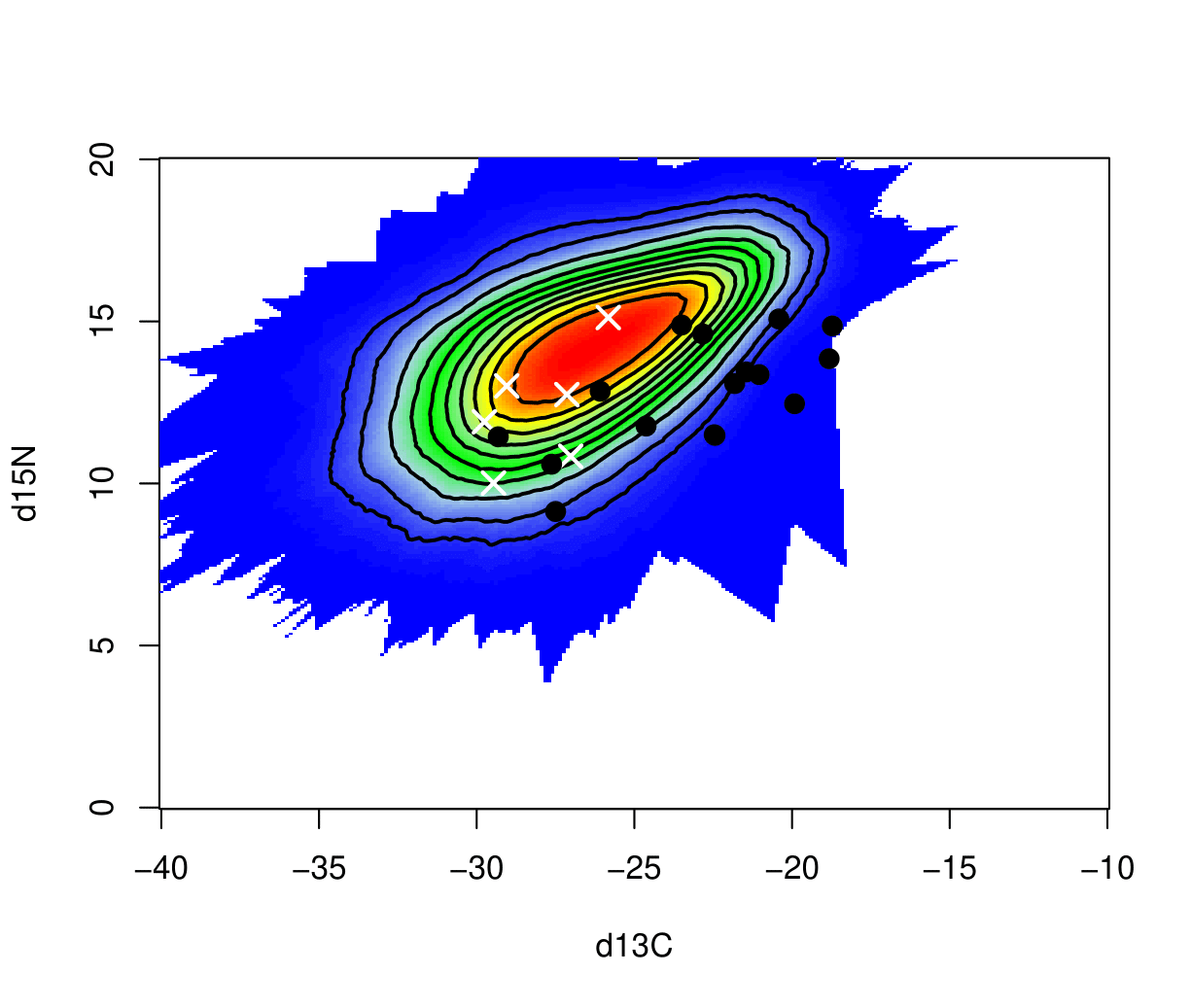


Supplemental Figure 2 Left: Raw isospace plot of fish species in Little Sturgeon Bay (lacustrine wetland). Species codes are indicated in Table 1. Right: The simulated mixing region for the biplot shown on the left.


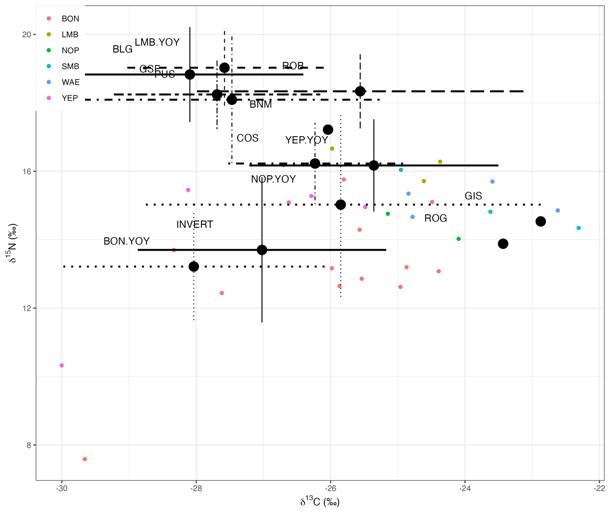

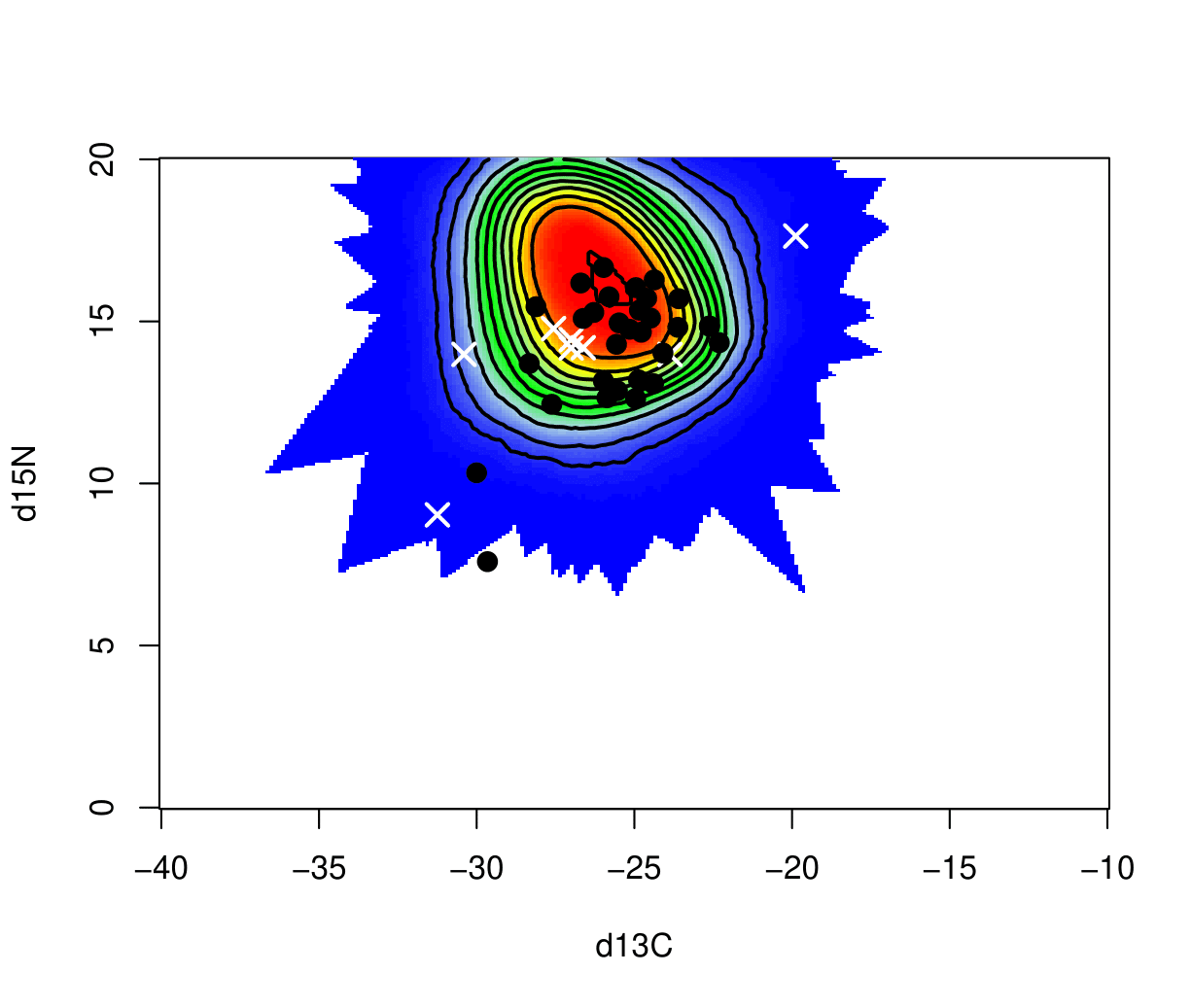


Supplemental Figure 3 Left: Raw isospace plot of fish species in Pensaukee River (riverine wetland). Species codes are indicated in Table 1. Right: The simulated mixing region for the biplot shown on the left.


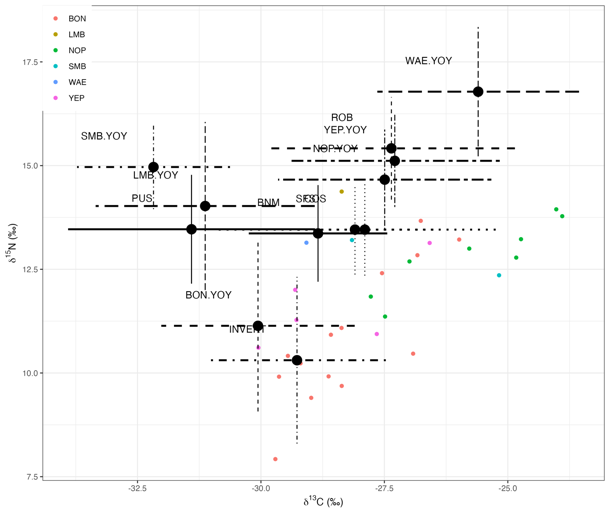

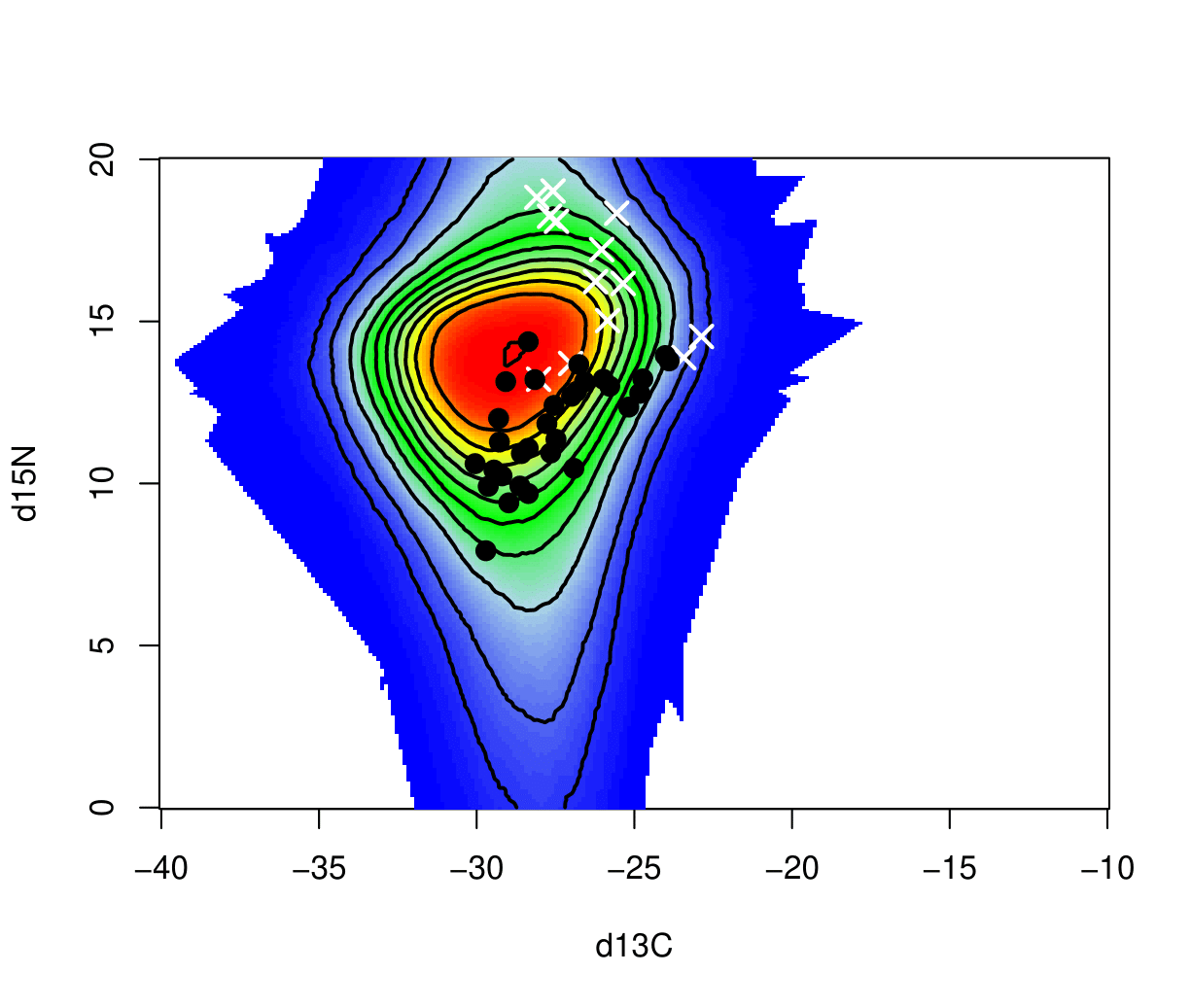


Supplemental Figure 4 Left: Raw isospace plot of fish species in Peshtigo River (riverine wetland). Species codes are indicated in Table 1. Right: The simulated mixing region for the biplot shown on the left.


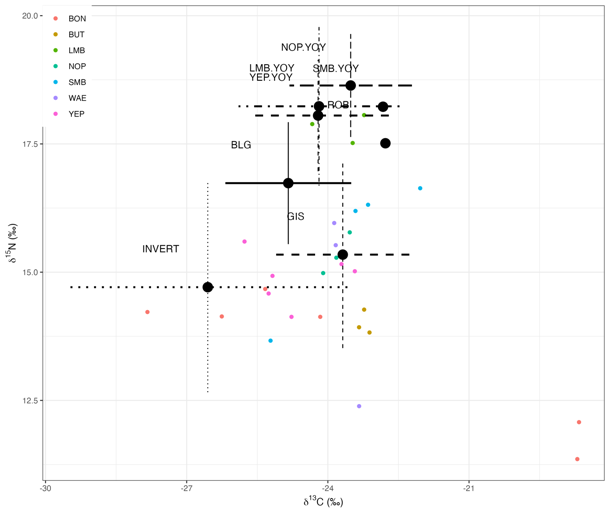

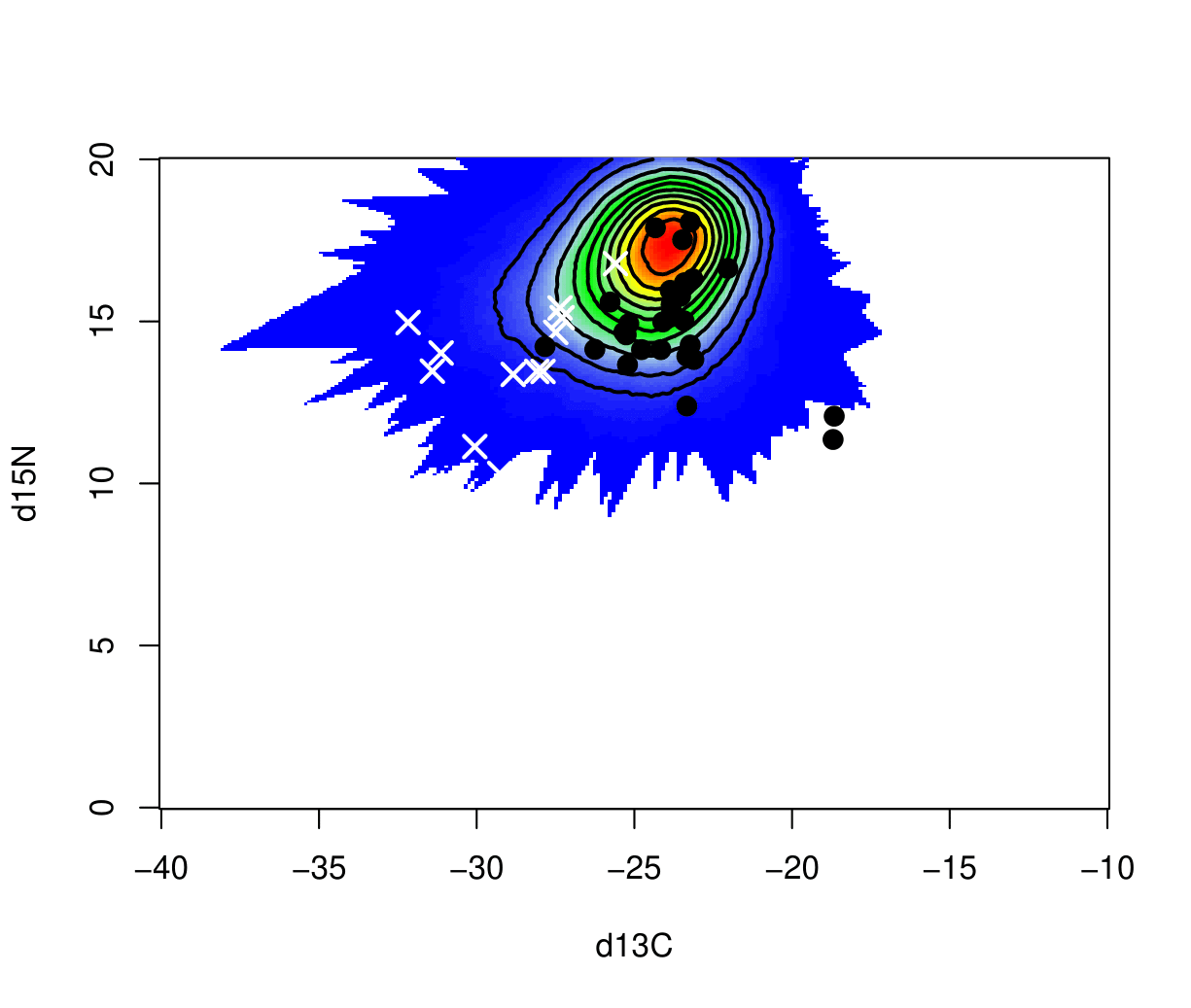


Supplemental Figure 5 Left: Raw isospace plot of fish species in Point au Sable (lacustrine wetland). Species codes are indicated in Table 1. Right: The simulated mixing region for the biplot shown on the left.


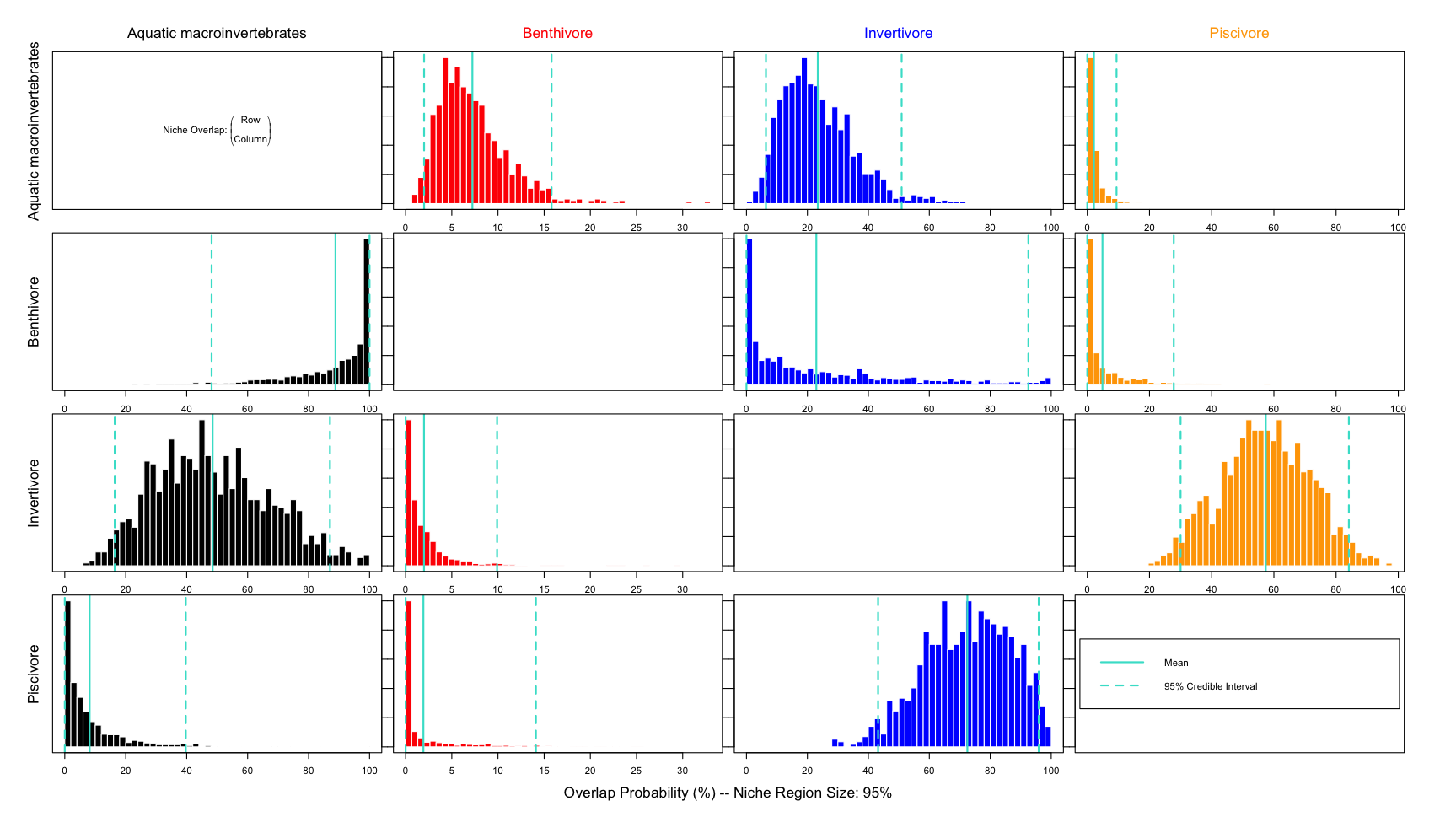


Supplemental Figure 6 Niche overlap estimates for 95% niche region sizes at Cedar River (riverine wetland).


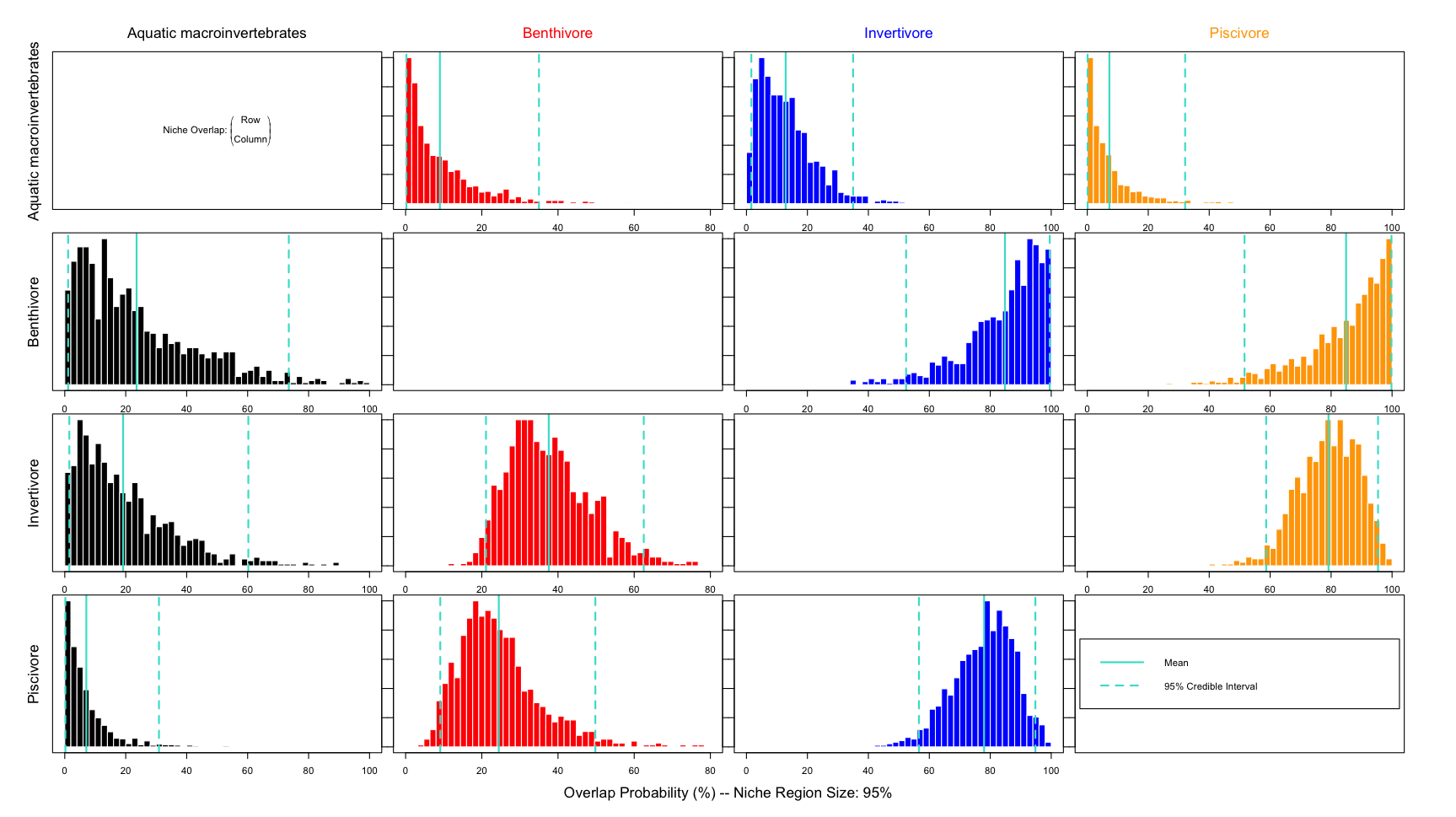


Supplemental Figure 7 Niche overlap estimates for 95% niche region sizes at Little Sturgeon Bay (lacustrine wetland).


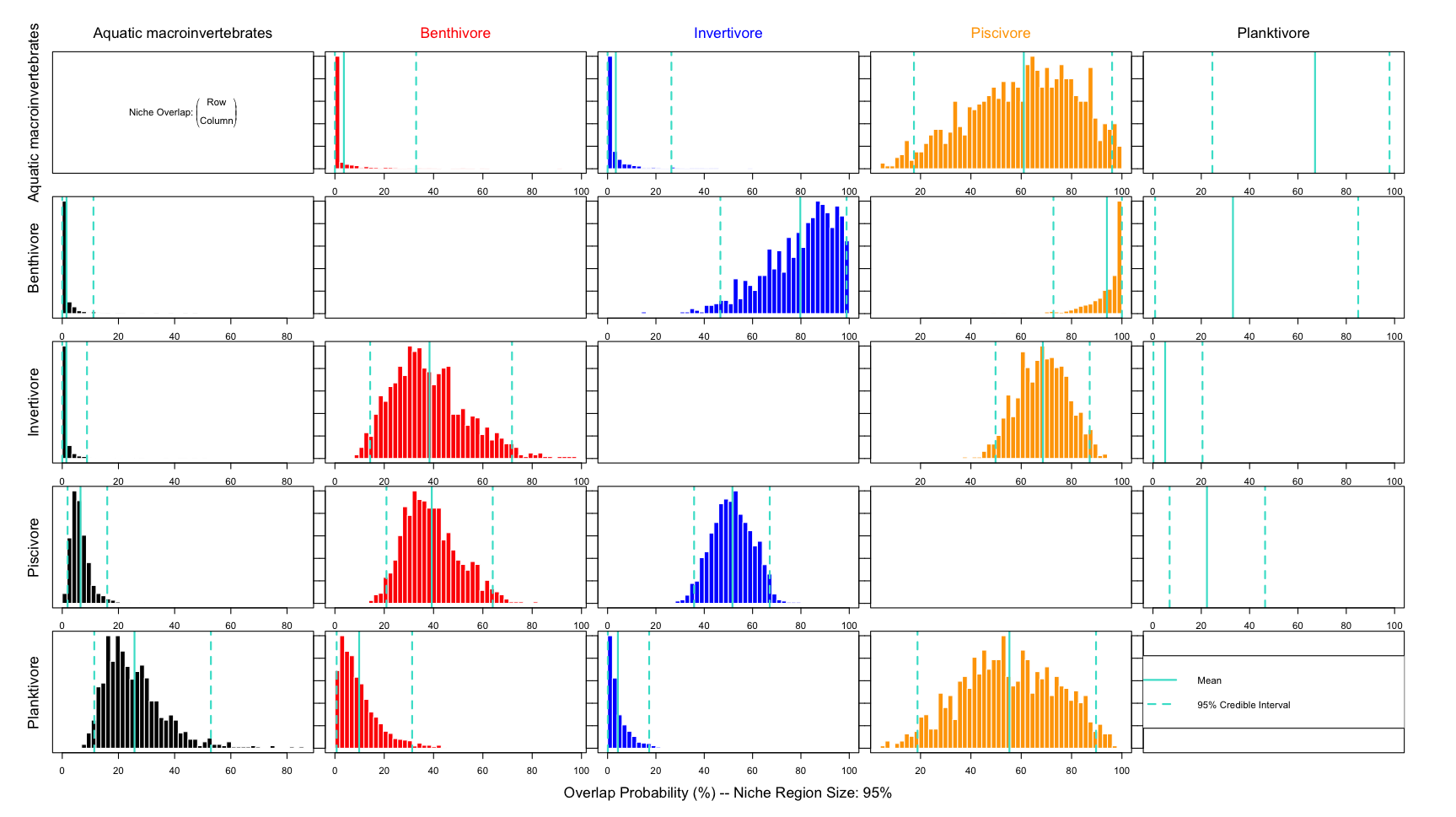


Supplemental Figure 8 Niche overlap estimates for 95% niche region sizes at Pensaukee River (riverine wetland).


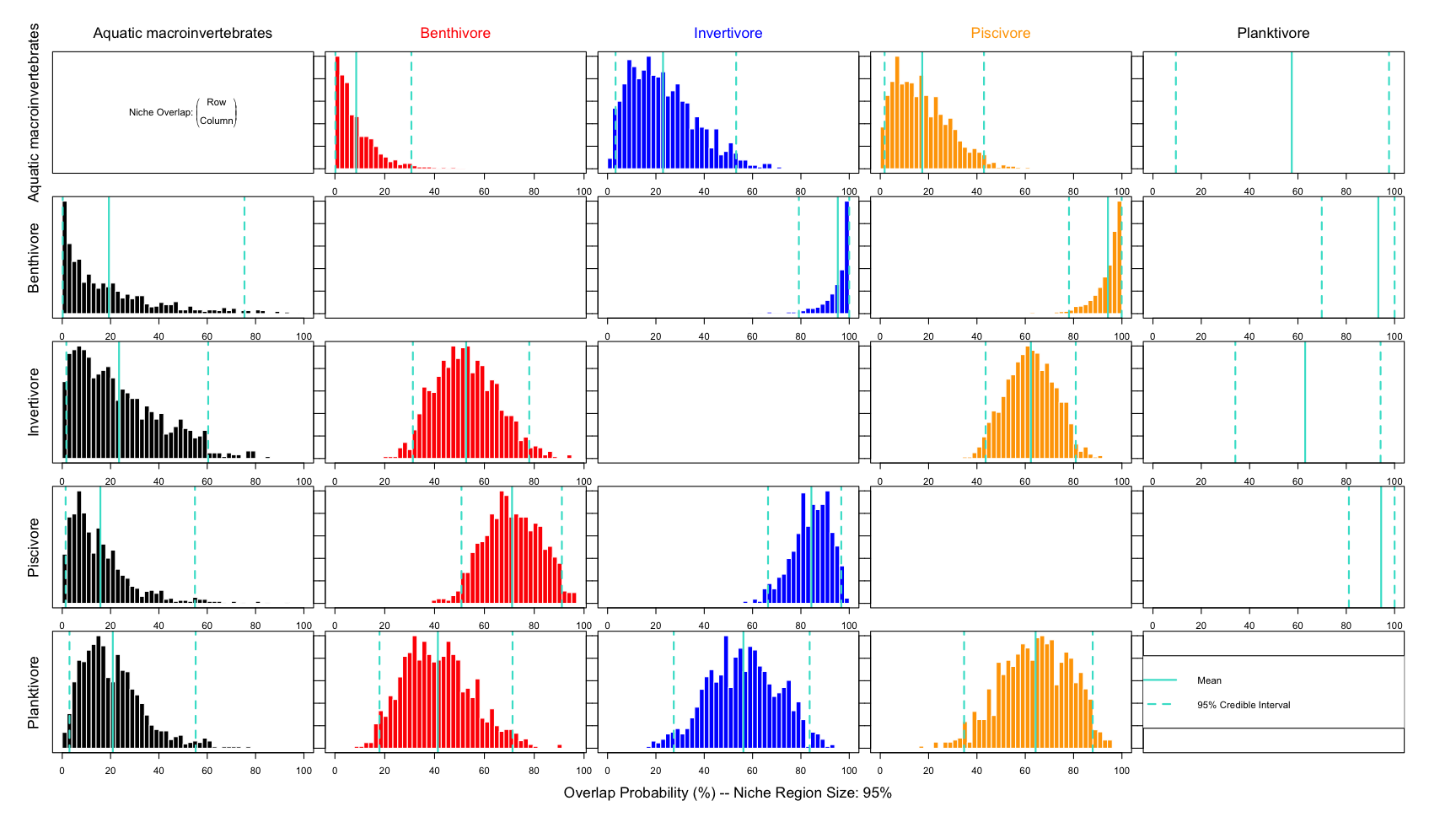


Supplemental Figure 9 Niche overlap estimates for 95% niche region sizes at Peshtigo River (riverine wetland).


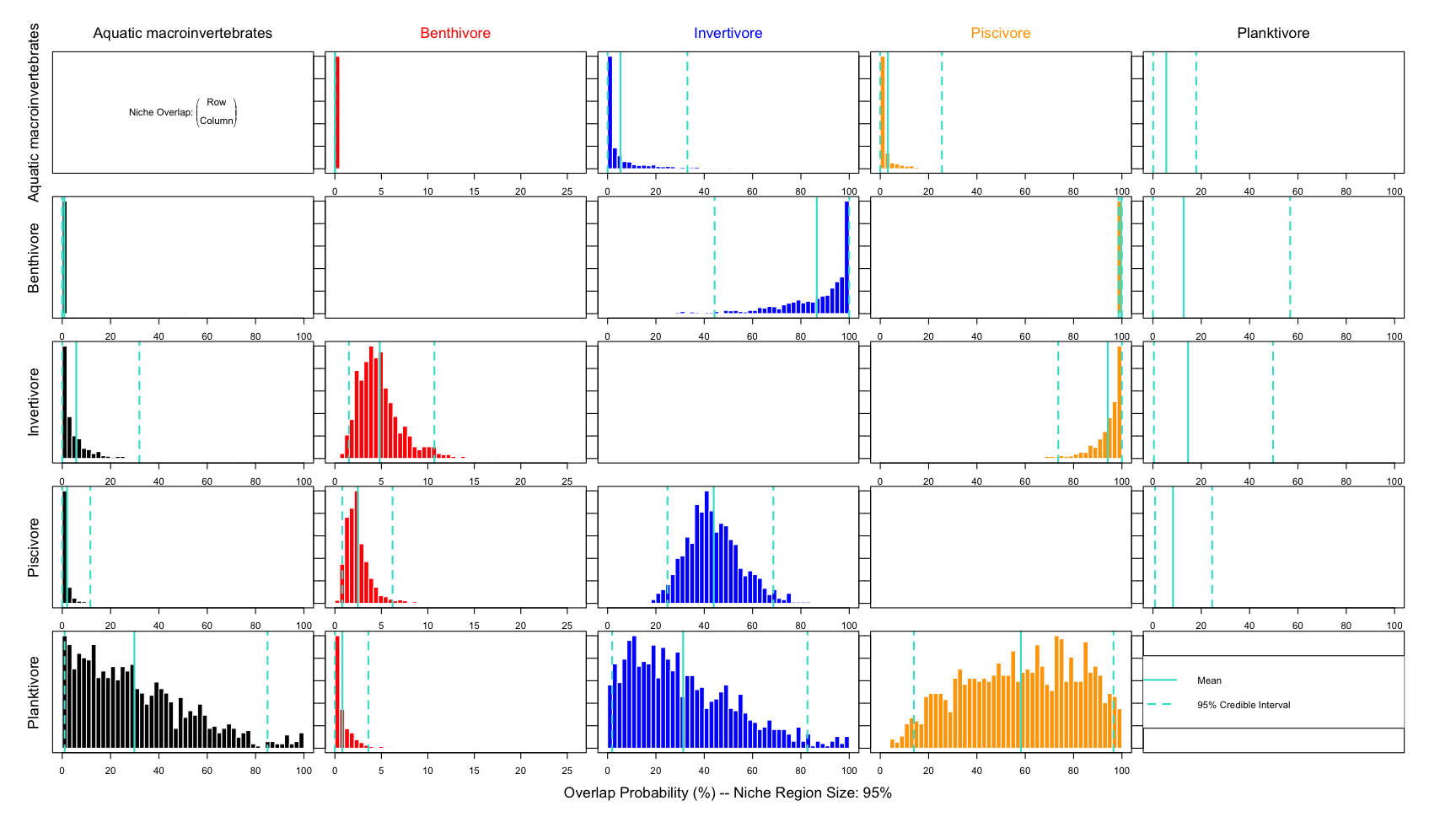


Supplemental Figure 10 Niche overlap estimates for 95% niche region sizes at Point au Sable (lacustrine wetland).


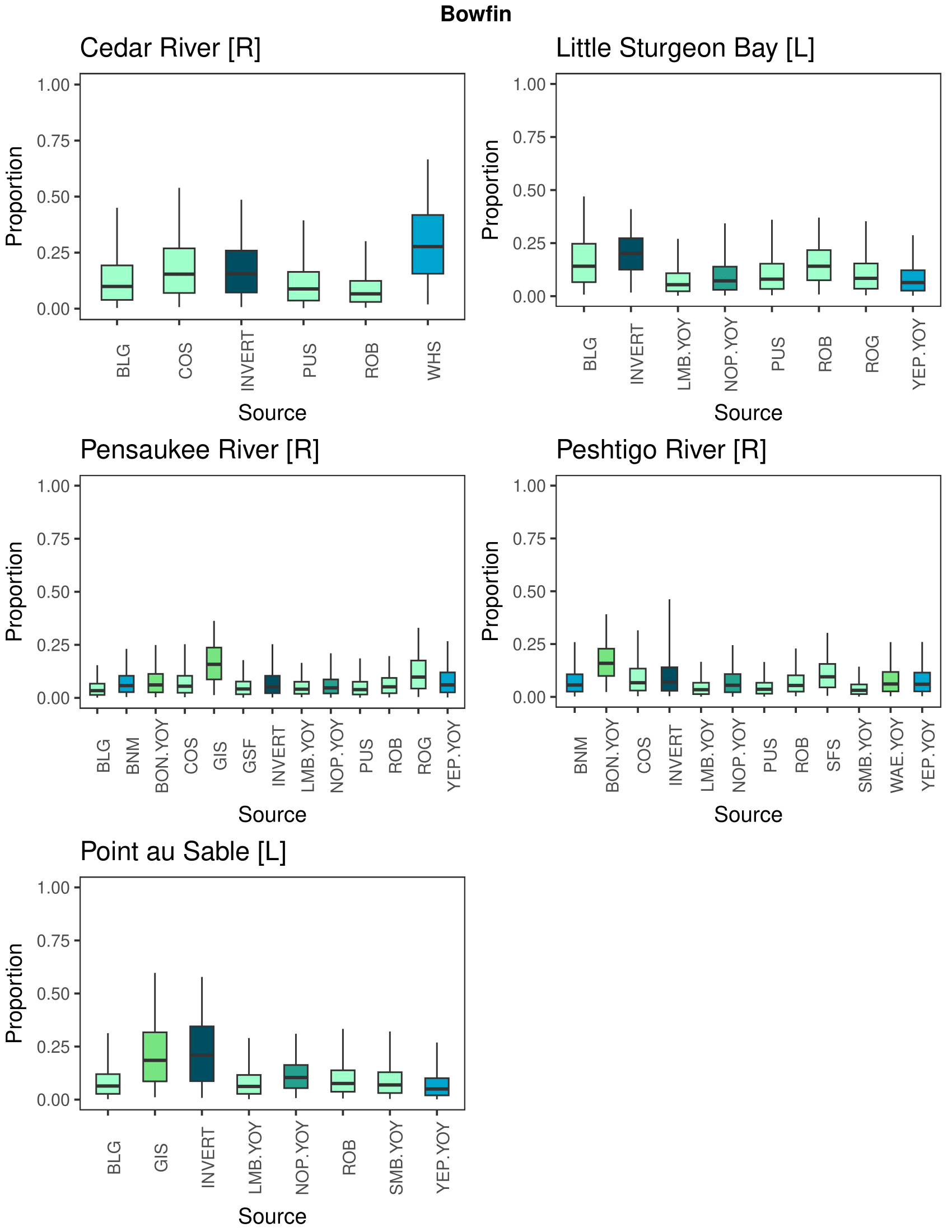

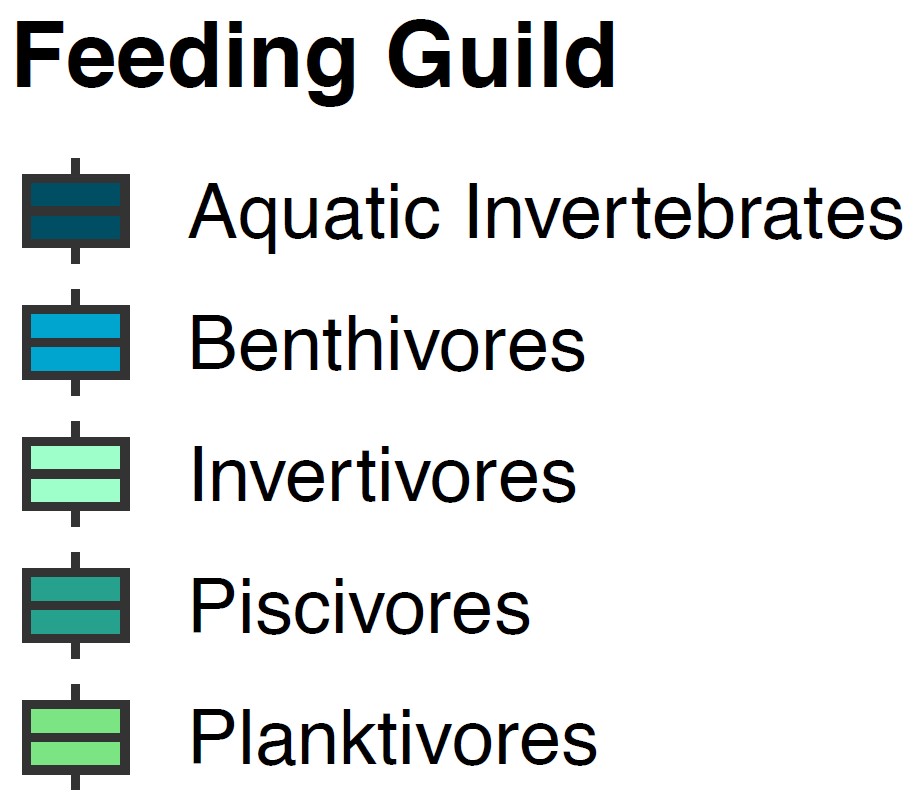


Supplemental Figure 11 Diet contribution estimates of bowfin *Amia calva* diet contributions from various feeding guilds at each wetland site. Higher percent contributions indicate a larger proportion of diet is comprised of that source (i.e., prey item). The horizontal black line represents the median posterior estimate, the box represents the upper and lower quartiles, and the vertical line represents the 95% credible interval. Species codes are indicated in Table 1.


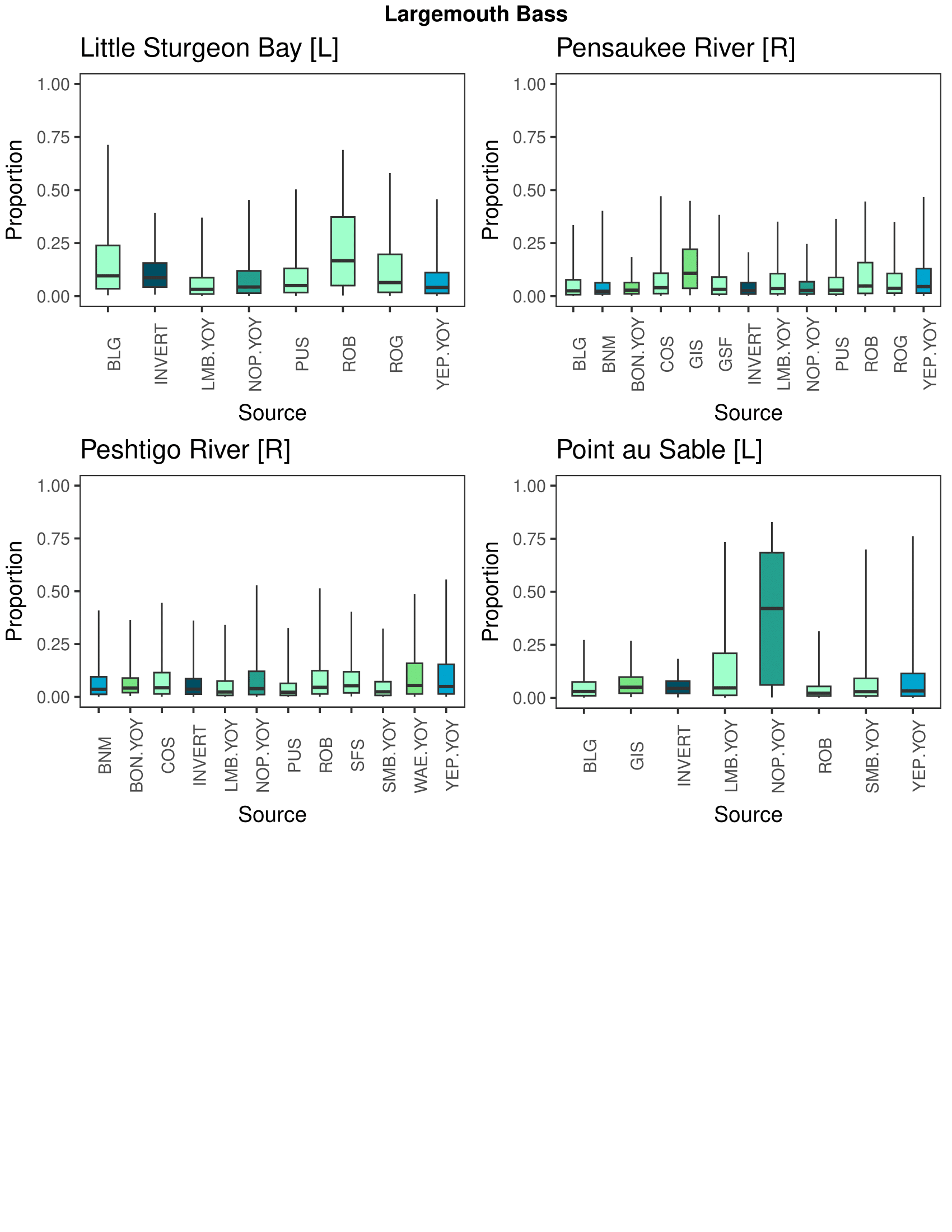


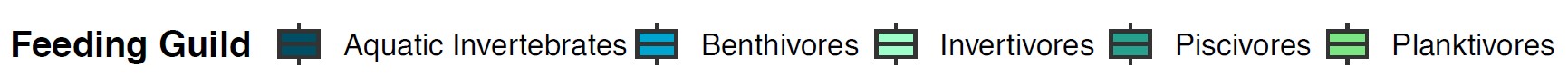


Supplemental Figure 12 Diet contribution estimates of largemouth bass *Micropterus salmoides* diet contributions from various feeding guilds at each wetland site. Higher percent contributions indicate a larger proportion of diet is comprised of that source (i.e., prey). The horizontal black line represents the median posterior estimate, the box represents the upper and lower quartiles, and the vertical line represents the 95% credible interval. Species codes are indicated in Table 1.


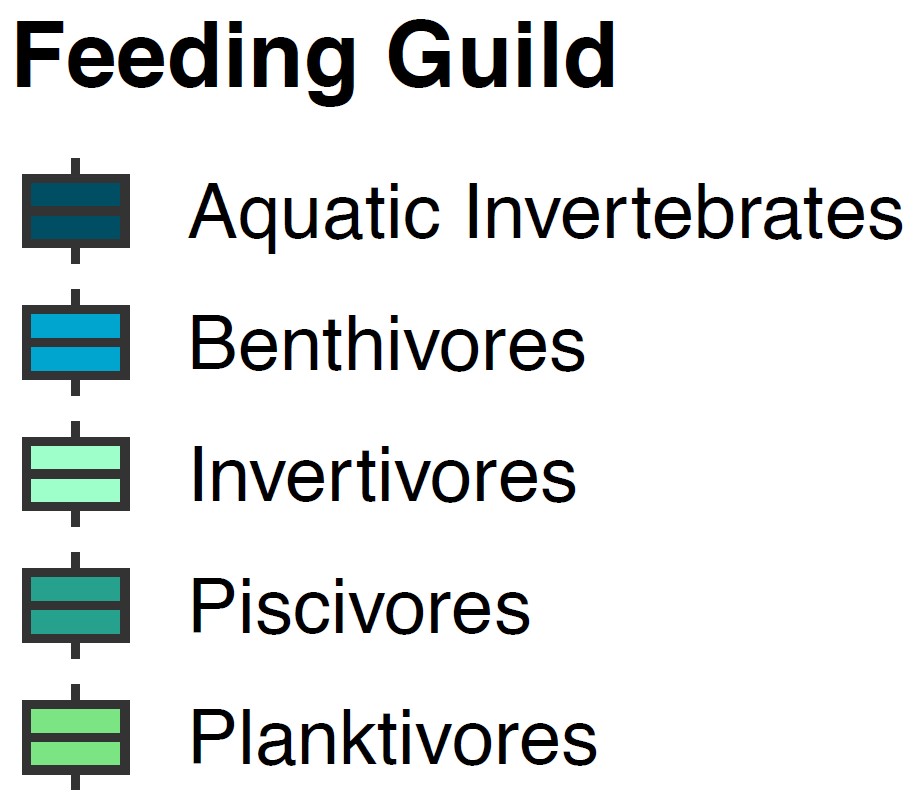

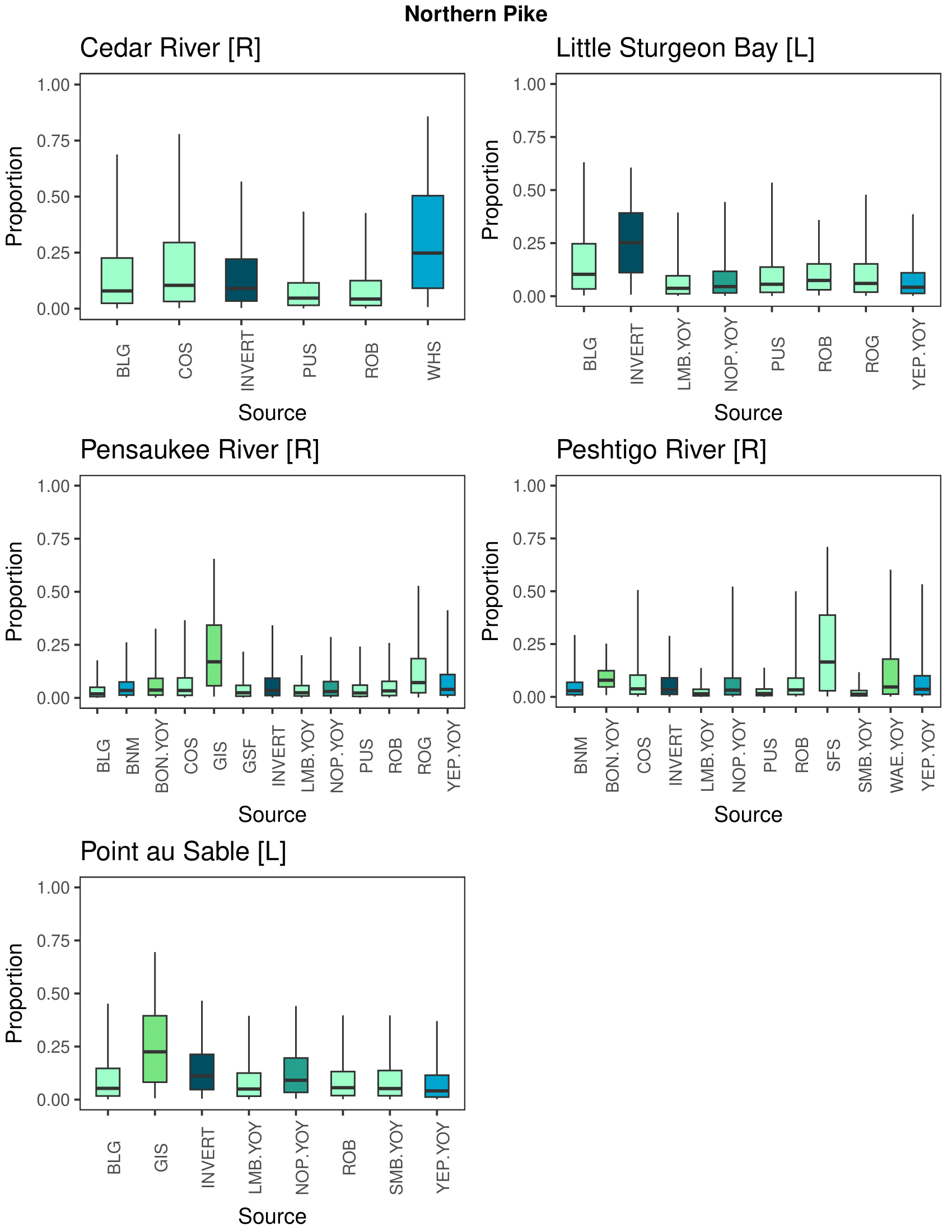


Supplemental Figure 13 Diet contribution estimates of northern pike *Esox lucius* diet contributions from various feeding guilds at each wetland site. Higher percent contributions indicate a larger proportion of diet is comprised of that source (i.e., prey). The horizontal black line represents the median posterior estimate, the box represents the upper and lower quartiles, and the vertical line represents the 95% credible interval. Species codes are indicated in Table 1.


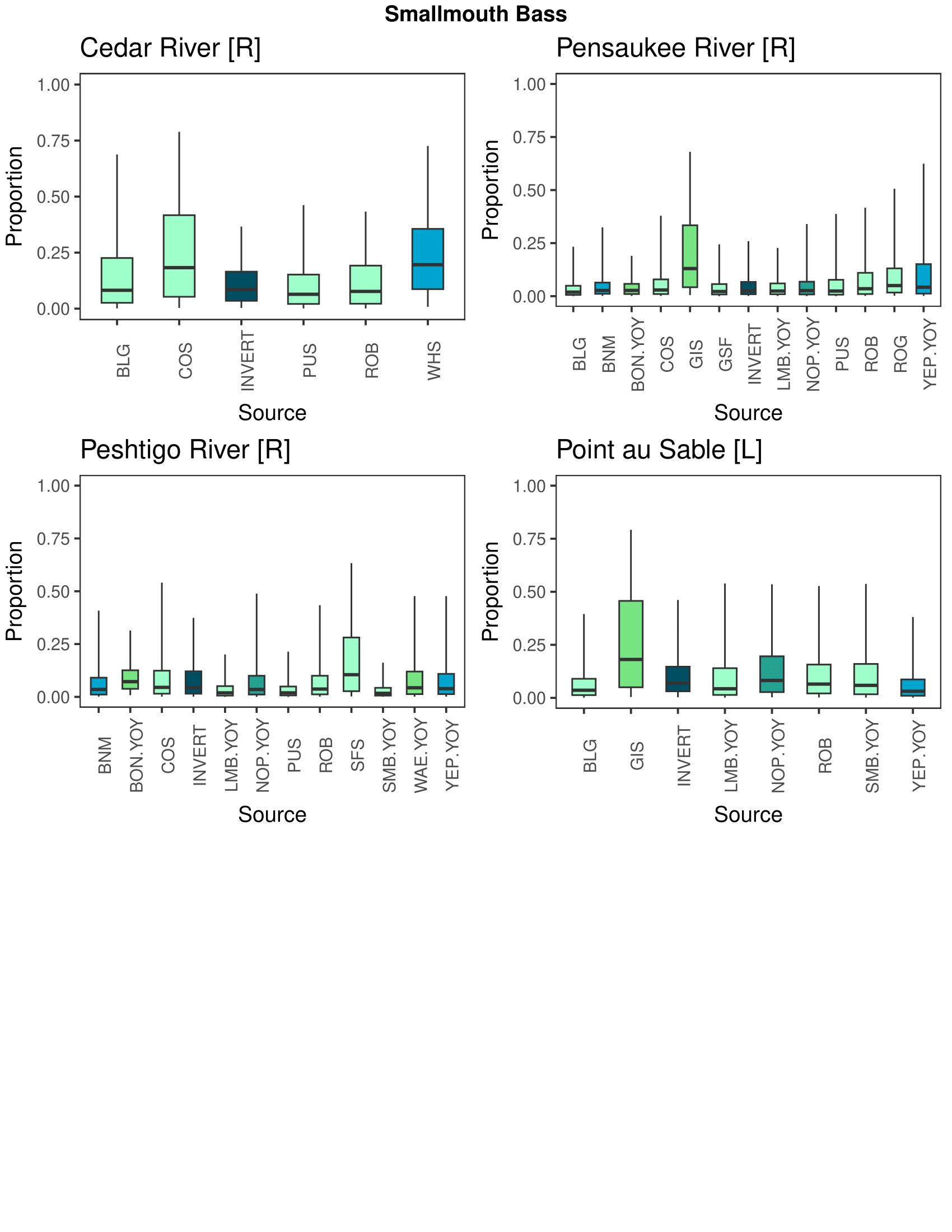


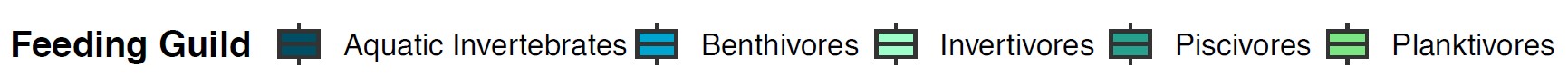


Supplemental Figure 14 Diet contribution estimates of smallmouth bass *Micropterus dolomieu* diet contributions from various feeding guilds at each wetland site. Higher percent contributions indicate a larger proportion of diet is comprised of that source (i.e., prey). The horizontal black line represents the median posterior estimate, the box represents the upper and lower quartiles, and the vertical line represents the 95% credible interval. Species codes are indicated in Table 1.


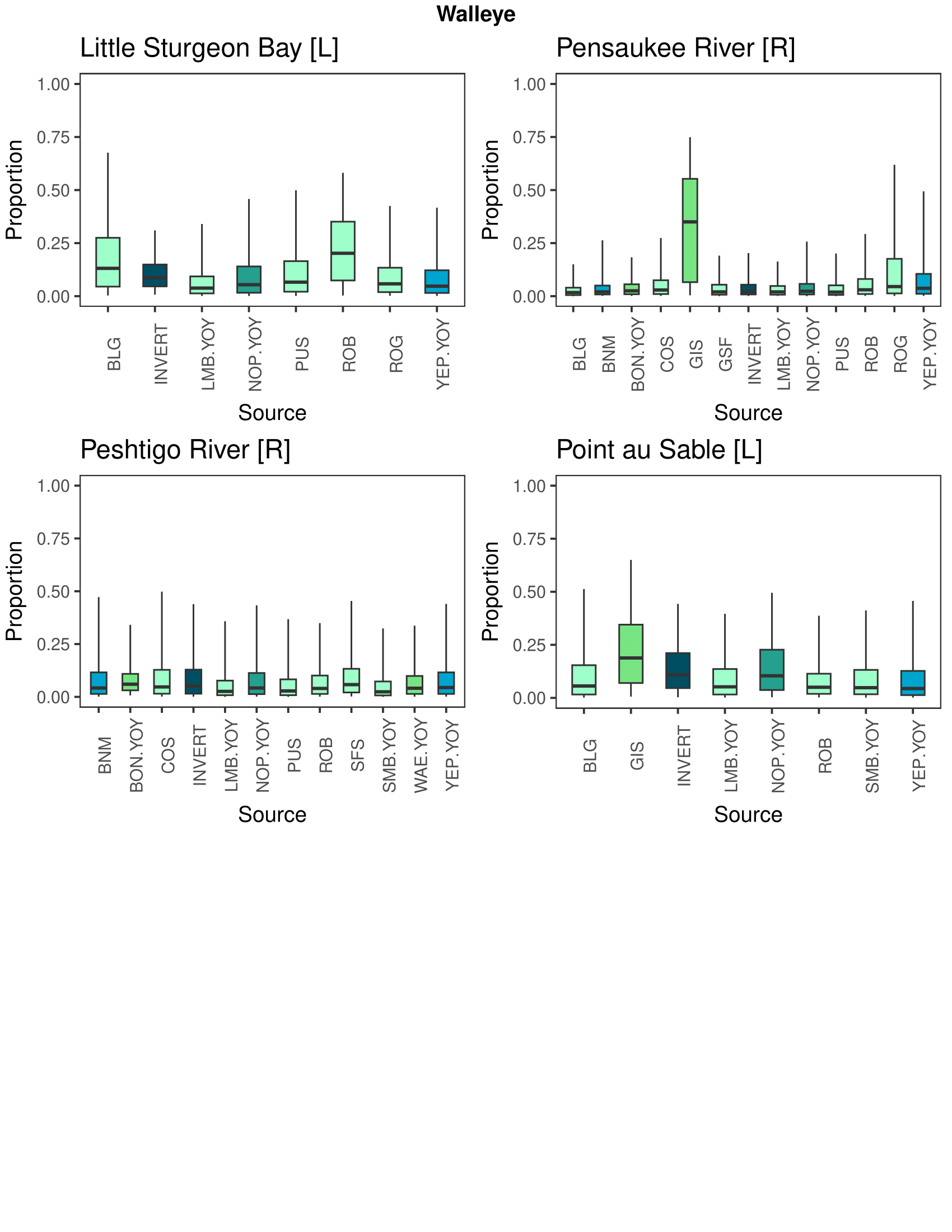


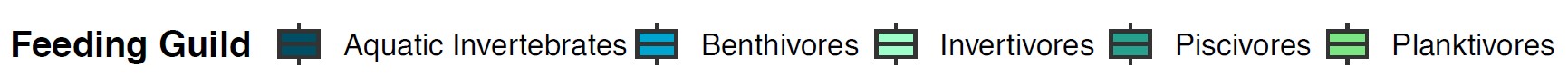


Supplemental Figure 15 Diet contribution estimates of walleye *Sander vitreus* diet contributions from various feeding guilds at each wetland site. Higher percent contributions indicate a larger proportion of diet is comprised of that source (i.e., prey). The horizontal black line represents the median posterior estimate, the box represents the upper and lower quartiles, and the vertical line represents the 95% credible interval. Species codes are indicated in Table 1.


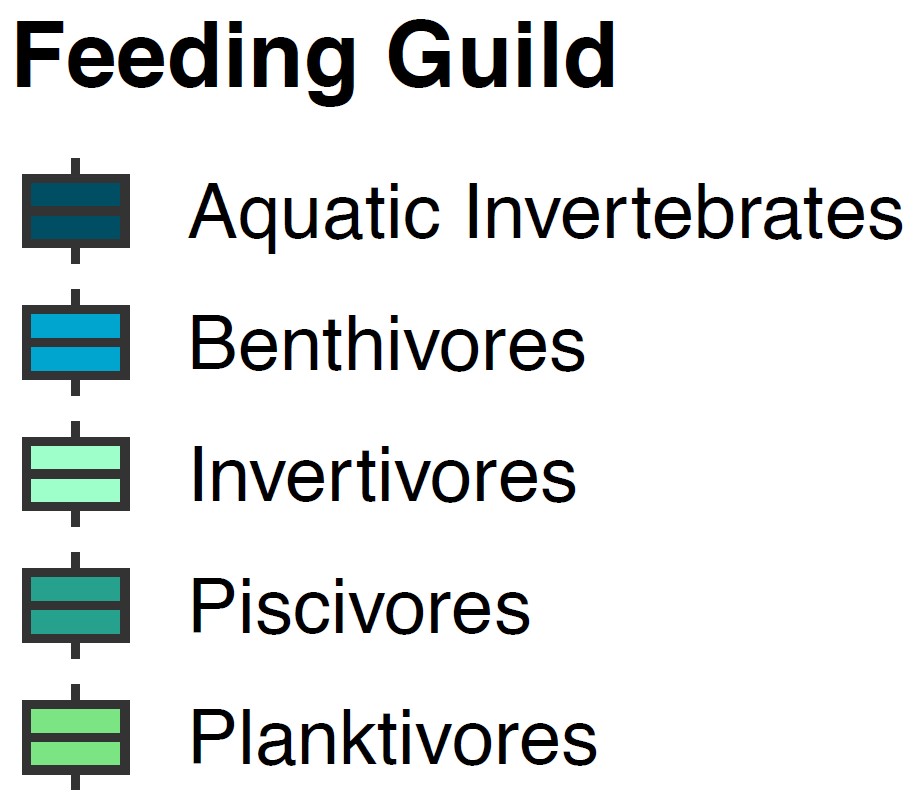

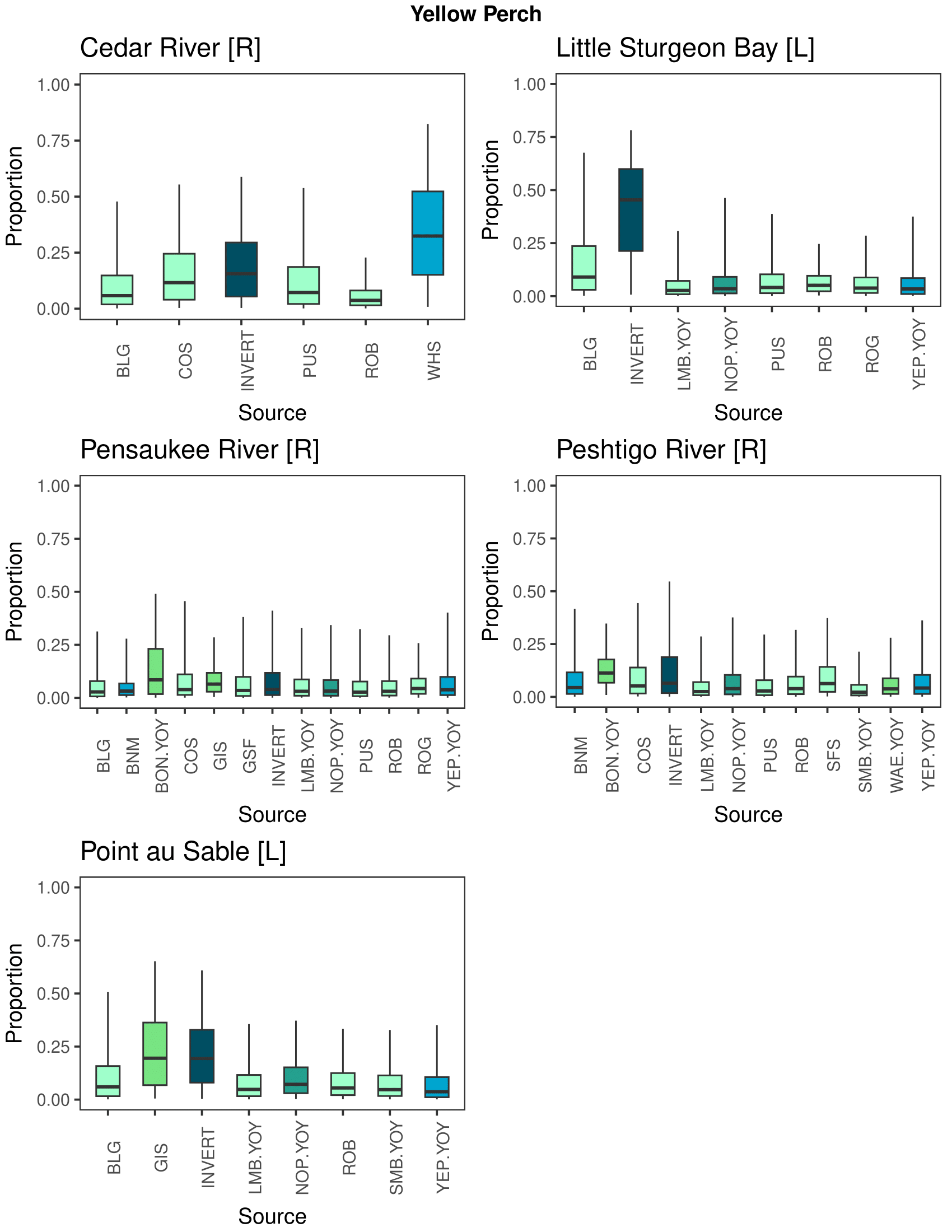


Supplemental Figure 16 Diet contribution estimates of yellow perch *Perca flavescens* diet contributions from various feeding guilds at each wetland site. Higher percent contributions indicate a larger proportion of diet is comprised of that source (i.e., prey). The horizontal black line represents the median posterior estimate, the box represents the upper and lower quartiles, and the vertical line represents the 95% credible interval. Species codes are indicated in Table 1.
